# Effect of Wine Yeast (*Saccharomyces* sp.) Strains on the Physicochemical, Sensory, and Antioxidant Properties of Plum, Apple, and Hawthorn Wines

**DOI:** 10.3390/foods14162844

**Published:** 2025-08-16

**Authors:** František Lorenc, Markéta Jarošová, Jan Bedrníček, Vlastimil Nohejl, Eliška Míková, Pavel Smetana

**Affiliations:** 1 Department of Food Biotechnologies and Agricultural Products’ Quality, Faculty of Agriculture and Technology, University of South Bohemia in České Budějovice, Studentská 1668, 370 05 České Budějovice, Czech Republic; bedrnicek@fzt.jcu.cz (J.B.); nohejl@fzt.jcu.cz (V.N.); mikove00@fzt.jcu.cz (E.M.); smetana@fzt.jcu.cz (P.S.); 2 Department of Plant Production, Faculty of Agriculture and Technology, University of South Bohemia in České Budějovice, Na Sádkách 1780, 370 05 České Budějovice, Czech Republic; jarosovam@fzt.jcu.cz

**Keywords:** fruit wine, yeast, sensory analyses, antioxidant activity, hawthorn, apple, plum

## Abstract

Fruit wines have become a popular alternative to grape wines for their variability of sensory properties and unique chemical profiles, offering interesting biological activities. Winemaking also utilizes fruits, which are usually sensitive to biological deterioration, thus reducing post-harvest losses. The quality of wines depends on the fermentation conditions, including the wine yeast selection. In this study, we observed the effect of three common *Saccharomyces* wine yeast strains on the physicochemical characteristics (color, pH, ethanol content), antioxidant potential (total polyphenol content—TPC, DPPH, and ABTS antioxidant assays), and sensory properties and their relations within plum, apple, and hawthorn wines. Generally, we observed quite-wide ranges in physicochemical properties (pH: 2.8–3.8, ethanol content: 9.0–16.2%) and antioxidant potential parameters (TPC: 0.5–2.4 mg/GAE, DPPH: 0.3–1.4 mg/AAE, 0.5–3.0 mg/AAE), which were affected by the fruit, yeast, and sampling term. The yeast strain significantly affected physicochemical properties and the antioxidant potential on a minor scale. The highest impact of yeast was observed within sensory analyses, where the hawthorn and apple wines fermented by yeast strain Fruit Red exhibited a different sensory profile than those fermented by the Buket and Special strains. A positive correlation between antioxidant potential parameters and their relationship with wine color was confirmed. Moreover, the overall acceptability grew with sweet taste intensity, and panelists preferred wines with lower ethanol content. In general, this study proved the significant impact of wine yeast strain selection on certain qualitative parameters of fruit wines.

## 1. Introduction

Wine is an alcoholic beverage produced from fermented fruit, typically from wine grapes (*Vitis vinifera*). Along with beer, wine from grapes represents the most popular alcoholic beverage. According to the International Organization of Vine and Wine, 237 million hectoliters of grape wine were produced worldwide in 2023 [1]. The wines produced from fruits other than wine grapes are typically referred to as fruit wines. Since some fruits are sensitive to microbial contamination and a decrease in sensory quality, alcoholic fermentation represents a way of fruit preservation, leading to reductions in post-harvest losses. The typical fruits used for wine production are cherry, apple, plum, blueberry, bilberry, peach, and mango [2].

Fruit wines are interesting alternatives to the common grape wine from more perspectives. They offer a wide variety of sensorial products, based on the type of original fruit. Since grape wine contains many bioactive compounds, especially polyphenols like resveratrol [3], different fruits provide various and sometimes unique compounds and bioactive properties. Therefore, different fruit wines may provide specific health-beneficial effects [4]. For instance, both jackfruit and custard apple wines demonstrate significant protective effects against DNA damage [5,6]. As reported by another study, mango wine may reduce cardiovascular risk by inhibiting LDL low-density lipoprotein oxidation [7]. Garcinia wine is recognized as a functional food with total polyphenol content and antioxidant activity [8]. Another study summarized that fruit wines made from dark small berries, such as blueberry, elderberry, and black currant, can have contents of phytonutrients, minerals, and antioxidants nearly as high as, equal to, or greater than red grape wines [9]. Furthermore, an in vivo study by Kim et al. [10] proved the anti-obesity and anti-atherogenic effects of natural tomato wine. An important advantage is the variability of fruit wine production. Many fruit types are more accessible and cheaper than wine grapes. The creative process of fruit winemaking allows producers to blend different fruit wines with each other or with grape wines [11].

In the Czech Republic, apples and plums are the most typical fruits used for fruit wine and distillate production. Along with these fruits, Chinese hawthorn becomes a popular and interesting alternative to the fruits typical of the Central European region. All three of these species are rich sources of bioactive compounds. Plums are characterized by high contents of phenolic compounds (mainly caffeic acid, chlorogenic acid, cryptochlorogenic acid, and neochlorogenic acid), anthocyanins, carotenoids, organic acids, minerals, and vitamins [12]. They also contain various organic acids, with malic acid being the dominant one, followed by quinic acids. The other acids present at a minor scale are tartaric, citric, oxalic, and succinic acids [13]. Apples are a rich source of selected micronutrients (e.g., iron, zinc, vitamins C and E) and polyphenols (e.g., procyanidins, phloridzin, neochlorogenic acid). They are also composed of a high extent of dietary fiber [14]. Similarly, like plums, apples’ predominant organic acid is malic acid, followed by citric acid. The other organic acids like quinic, succinic, shikimic, oxalic, and tartaric acids are present to a much lower extent [15,16]. Chinese hawthorn berries contain polyphenols (flavonoids, proanthocyanidins, and phenolic acids like gallic, caffeic, syringic, neochlorogenic, and cryptochlochlorogenic acids) and soluble dietary fiber (mainly pectin) [17]. Other bioactive components of hawthorn fruit are terpenoids, lignans [17], and vitamins C and B2 [18]. The dominant organic acids of Chinese hawthorn fruits are oxalic, tartaric, malic, shikimic, and citric acids. According to the cited study, citric acid was strongly dominant, followed by malic acid, while the content of other named acids was much lower [19].

The quality of fruit wine can be affected by many factors such as the plant genotype determining the chemical composition of fruit, post-harvest treatment, and production technology [2]. Selecting yeast species or strains as starter cultures is one of the most important steps in wine production technology as it plays an important role in defining wine’s sensorial identity [20]. Sensory and organoleptic properties of wine are predominantly determined by the contents of various fermentation byproducts, such as glycerol, carboxylic acids, esters, aldehydes, and sulfides or higher alcohols formed by the degradation of sugars, fatty acids, amino acids, terpenes, and thiols, whereas these properties and related processes largely depend on the characteristics and activity of the starter yeasts [21]. There are two groups applicable for fruit wine fermentation: *Saccharomyces*, simply divided into the species *S. cerevisiae*, and *S. bayanus*, which are further classified into various races and subspecies. Different species of *Saccharomyces* yeast can have different impacts on the properties of the produced wine. One study compared the effect of *S. cerevisiae*, *S. bayanus,* and *S. pastorianus*; the last species is non-typical for winemaking and is rather used for beer production. This study reported that the mixed culture of *S. pastorianus* and *S. bayanus* produced grape wines with a higher intensity of citrus fruit notes than the wines fermented by the commercial *S. cerevisiae* strains. It also suggested that the inoculation conditions can modulate the yeast’s performance [22]. Another study compared *S. cerevisiae* and the closely related species *S. paradoxus*, which exhibited higher glycerol and lower acetic acid production than *S. cerevisiae* in a model wine system, thus highlighting its interesting enological properties [23]. Other studies compared the effects of different *S. cerevisiae* strains. One of the studies found differences in the grape wines fermented by two strains, specifically in the levels of alcohol and esters [24]. Another, more developed, study observed the metabolomic signature of grape wines fermented by twelve *S. cerevisiae* strains. It reported a wide range of non-volatile metabolites of different types of compounds, such as carbohydrates, polyphenols, lipids, peptides, aminosugars, and their derivatives. It also confirmed that each strain can be associated with a specific metabolomic fingerprint [25]. The significant differences between *S. cerevisiae* strains were confirmed also in a greengage alcoholic beverage, where using four different strains led to the production of beverages with aromatic compounds compositions and profile characteristics. These differences were mostly attributed to β-ionone and ethyl octanoate [26]. The other yeasts are represented by a group of non-*Saccharomyces* represented by various genera and species (e.g., *T. delbrueckii*, *M. pulcherrima*, *H. uvarum*, *L. thermotolerans*, *W. anomalus*, *P. kluyveri*, *H. thailandica*, *C. zemplinina*, *S. pombe*) [2,27]. Using non-*Saccharomyces* yeasts or mixed-type cultures of yeasts to ferment fruits can be applicable to obtain fruit wines with specific and atypical physicochemical and chemical compositions or bioactive properties. It has been reported that fruit wines produced by pure non-*Saccharomyces* or mixed-type (*Saccharomyces* + non-*Saccharomyces*) cultures can have different traits compared to wines fermented by pure *Saccharomyces* yeasts such as increased pH, decreases in the levels of ethanol and higher alcohols, and eventual changes in bioactive compound contents (organic acids, polyphenols, terpenes, esters) and antioxidant activity [2].

Fruit wines can be interesting for consumers mainly from the point of two factors: sensory parameters and potential health benefits. Home and small-scale producers of fruit wine typically use the commonly accessible wine yeast strains. Therefore, it is important to study the effect of these strains on the properties of typically made or popular fruit wines. This work aimed to determine and compare the effects of three different common strains of *Saccharomyces* wine yeasts on the pH, ethanol content, color, and development of the total polyphenol content and antioxidant activities (via scavenging DPPH and ABTS radicals) during the production process and the sensory properties of plum, apple, and hawthorn wines.

## 2. Materials and Methods

### 2.1. Materials

In this study, three types of fruit were used for wine production. As the pome fruits were used apples of two cultivars (*Malus domestica* cv. ‘Šampion’ and ‘Rubinola’; mixture 1:1 *w*/*w*) and Chinese hawthorn (*Crataegus pinnatifida* cv. ‘Big Mao Mao’). As the stone fruit were used plums (*Prunus domestica* cv. ‘Haganta’). Apples and plums are traditionally used for fruit winemaking in the Czech Republic while hawthorn berries are rarely grown and processed and are not used for winemaking. They were selected to compare determined properties among them and explore the potentially different impacts of various yeast strains on these diverse types of fruits For the fermentation, three commonly used strains of wine yeasts lyophilized were used, obtained from the e-shop “Vinařský dům Kopeček” (Czech Republic), with different oenological characteristics, thus providing potentially different sensory or other determined properties of the three types of fruit wines. Three wine yeasts typically used for the production of Central European grape wines were represented by FermiHill Special (*Saccharomyces bayanus*, strain ‘Fermivin LS2’) used for primary and secondary fermentation, suitable to produce sparkling wines and ciders; FermiHill Fruit Red (*Saccharomyces cerevisiae*, strain ‘Fermivin E73’), allowing to obtain fruity wines with notes of red fruits and flowers, characterized by high ester production; and FermiHill Buket (*Saccharomyces bayanus*, strain ‘Fermivin 4F9’), producing wines of very fruity notes with an emphasis on diverse and delicious aromas and a balanced taste with a long persistence. All these wine strains were originated and validated by Oenobrands SAS (Montferrier-sur-Lez, France), pectolytic enzyme “Distizym FM TOP” (ERBSLÖH Geisenheim, Geisenheim, Germany), and yeast nutrition “Complete yeast nutrients” (Gert Strand AB, Oxie, Sweden).

### 2.2. Winemaking Process

#### 2.2.1. Plum Wines

As the input resource, 25 kg of plums was processed to produce fruit wine. Firstly, the plums were cleaned and washed to remove any impurities. Then, the plums were destemmed and pitted. Pitted plums were then juiced using a commercial juice extractor Catler JE 4011 (FAST ČR a.s., Říčany, Czech Republic). Due to the high foaming capacity, the obtained must was left in the cold for 24 h. Then, 1 g (per 4 liters of must) of dehydrated yeast of each strain, namely Buket (B), Fruit Red (F), and Special (S), was weighed and rehydrated in ten times the volume of the mixture from plum juice and lukewarm water (1:1). Solubilized yeasts were gently shaken and activated at 20 °C for 30 min. In the meantime, the sugar content of the untreated plum was measured using a portable refractometer, and the value was 23.0 °Brix. The amount of sugar contained in the fruit was sufficient, and it was not desirable to further adjust it. 9.6 g of yeast nutrition was added (0.8 g per L of must), and 1.8 mL (empirically reflecting the pectin content and the dose recommended by the manufacturer) of pectolytic enzyme was added to 12 L of must. Then, the plum was transferred equally into three 5 L plastic containers, so the total must volume in each container was 4 L. The containers were fitted with a lid and a fermentation stopper filled with water. Subsequently, each container was marked with the appropriate type of yeast, and the yeast was mixed in the volume. The containers with fermented plum must were placed in a climate chamber, where they were tempered for nine days at 20 °C. The pH value was measured during fermentation on days 0 (fresh—before fermentation), 1, 2, 3, 5, 7, and 9. Samples of approximate volume between 10 and 12 mL were taken and frozen in 15 mL centrifuge tubes for later determination of antioxidant potential and color parameters. On day 9, the fermentation was finished, and the °Brix value was also recorded. On the ninth day after the start of fermentation, each variant of fruit wine was quickly tested to identify any serious sensory deficiencies that were not present. The wines were then carefully racked by the silicon food-grade hose, to avoid the transfer of yeast and solid fruit parts sitting on the bucket bottom, into the new clean bucket, followed by pouring into 0.5 L bottles with patent caps. More than four full bottles were obtained from each variant of unfiltered plum wine. These bottles were submerged in a pot with water, and the pasteurization was performed. During this procedure, the bottles containing wine were heated to a temperature of 60 °C, at which point mild pasteurization took 15 min. These conditions were selected as the established procedure used within our department for beer and wine pasteurization. This method was adopted from the study of Milani & Silva [28], who stated that 15 PU, corresponding to pasteurization for 15 min at 60 °C, is recommended for beer pasteurization, sufficient to inactivate the fermenting yeast and prevent undesirable contamination by microorganisms. Pasteurized wines are further in the manuscript described as “P” and were subjected to all physicochemical analyses. A gentle temperature and pasteurization time were chosen to preserve the original sensory parameters of the wine. The pasteurized bottles were then stored at 2 °C for subsequent sensory analysis.

#### 2.2.2. Apple Wines

The whole winemaking process to obtain apple wines was identical to the procedure described in Section 2.2.1, with the following distinctions. A quantity of 30 kg of apples was showered, crushed by a fruit crusher, and pressed by a fruit press to obtain 16 L of must. The original sugar content corresponded to 13 °Brix, so 2.1 kg of sugar was added to the whole must volume to achieve 23 °Brix to standardize the sugar content to the input °Brix value of plum must. Further, 12.8 g of yeast nutrition (0.8 g per L of must) and 2.4 mL (empirically reflecting the pectin content and the dose recommended by the manufacturer) of pectolytic enzyme were added. More than five full 0.5 L bottles with patent caps were obtained from each variant of apple wine.

#### 2.2.3. Hawthorn Wines

The whole winemaking process to obtain hawthorn wines was identical to the procedure described in Section 2.2.1, with the following distinctions. Due to the high pectin content in hawthorn berries, fruit processing was more complicated than in the case of plums and apples. Over 7 kg of hawthorn berries was washed and homogenized by hand blender into a very dense mash. Therefore, this mash was mixed with the boiled and cooled tap water in a ratio of 1:3 (fruit/water) to obtain 26 L of thick must. The original sugar content corresponded to 3.5 °Brix, so 3.9 kg of sugar was added to the whole must volume to achieve 23 °Brix to standardize the sugar content to the input °Brix value of plum must. Further, 20.8 g of yeast nutrition (0.8 g per L of must) and 5 mL (empirically reflecting the pectin content and the dose recommended by the manufacturer) of pectolytic enzyme were added. More than five full 0.5 L bottles with patent caps were obtained from each variant of hawthorn wine.

### 2.3. Wine Color and pH Measurements

**Figure 1:**
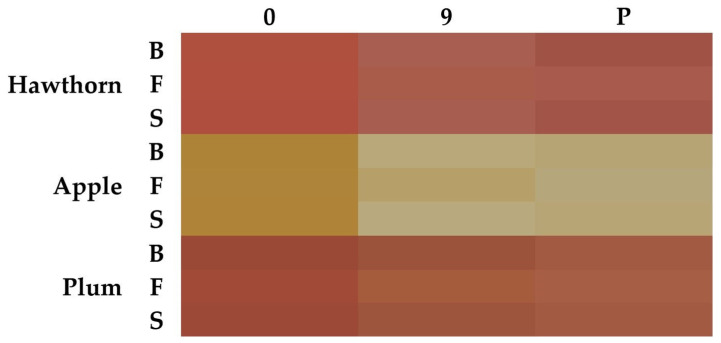
Color visualization (L = lightness, a = redness, b = yellowness) of hawthorn, apple, and plum wines (*n* = 3) fermented by different yeast strains on the zeroth and ninth days and after pasteurization (P)—; B: yeast “Buket”, F: yeast “Fruit Red”, S: yeast “Special”.

Color was measured within wines of all fruit and yeast variants after zero and nine days of fermentation and after pasteurization using a portable spectrophotometer, CM-25d (Konica Minolta, Tokyo, Japan), within the CIELAB color space (L*a*b*) and SCI mode. The visualization of true colors of certain wines (Figure 1) was performed by compiling L*, a*, and b* components using software SpectraMagic NX Pro Version 3.4 (Konica Minolta, Tokyo, Japan). pH of all variants of wines and all samplings was determined by pH meter GMH3531 (GHM—GREISINGER, Prague, Czech Republic).

### 2.4. Ethanol Content Determination

Alcohol (ethanol) content was determined by pycnometry according to the modified AOAC Official Method 942.06 [29]. Prior to distillation, 10 mL of 1N NaOH was added to 100 mL of the wine sample to precipitate volatile organic acids and prevent their distillation. Consequently, more than 80 mL of wine samples was distilled and collected into a 100 mL volumetric flask. A volumetric flask was filled with water to mark. The density of the diluted distillate was determined using a 50 mL Gay-Lussac pycnometer by weighing the pycnometer with the sample, the empty pycnometer, and the pycnometer filled with water using an analytical balance. The density of the sample was then calculated by the following formula:

$$h=\frac{m-y}{z}$$

Here, *h* represents the density of distillate (g/mL), *m* is the weight of the pycnometer with distillate (g), *y* is the weight of the empty pycnometer (g), and *z* is the weight of deionized water in the pycnometer (g). The relative ethanol content (% *v*/*v*) was determined according to the density of the final sample using the calculation table (Figure S1).

### 2.5. Sensory Analyses

The sensory profile of the nine fruit wine variants (3 fruits × 3 yeast strains) was tested by sensory analysis according to the modified method by Bedrníček et al. [30]. The sensory analyses were performed by 26 panelists, comprising 15 females and 11 males (23–61 years old), who had been well trained in the principles and the concept of sensory evaluation. The panelists were also informed about the alcohol content in the samples. Each panelist received 5 cl of wine samples in shot glasses. All nine samples were given to the panelists at the same time and were tasted only once, within one sensory analyses. The glasses were labeled by randomly selected two-digit codes chosen for each wine variant and served to panelists to compare them simultaneously. The hedonic evaluation of color, turbidity, aroma, overall taste, sour taste intensity, sweet taste intensity, and overall acceptability was recorded by panelists on a 10 cm unstructured hedonic scale (0 = unpleasant, 10 = very pleasant for color, taste; 0 = without turbidity, 10 = very turbid for turbidity; 0 = unacceptable, 10 = excellent for aroma and overall acceptability; 0 = undetectable, 10 = very sour for sour taste intensity; 0 = undetectable, 10 = very sweet for sweet taste intensity). The values for all descriptors of fruit wines from each panelist were averaged (n = 26). Water served as a taste neutralizer and was consumed by panelists between evaluating the following samples.

### 2.6. Total Polyphenol Content

To determine total polyphenol content (TPC) and antioxidant activities, all variants of wines sampled during fermentation and after pasteurization were used. Before the analyses, samples were centrifuged for 10 min at 4500 rpm, and the supernatant was used for the following assays.

TPC was determined using the Folin–Ciocâlteu reagent according to the method described in Jarošová et al. [31]. Reaction mixture was prepared by mixing 10 μL of extract with 990 µL deionized water and 50 μL of Folin-Ciocâlteu’s phenol reagent. After gentle shaking of the mixture, 150 µL of 20% sodium carbonate was added, and the samples were then incubated for 120 min at room temperature. Absorbance was measured at λ = 765 nm using a GENESYS 180 Double Beam UV-Vis spectrophotometer (Thermo Fisher Scientific, Waltham, MA, USA). A standard calibration curve was prepared with gallic acid at 0 to 2000 µg/mL concentrations. The obtained values of TPC were reported in milligrams of gallic acid equivalents per 1 mL of wine (mg GAE/mL). The mixture prepared for the reaction without a sample was used as a blank.

### 2.7. Antioxidant Activity

The antioxidant activity of fruit wine samples was determined via methods of scavenging activities against DPPH (2,2-Diphenyl-1-picrylhydrazyl) and ABTS (2,2′-Azino-bis(3-ethylbenzothiazoline-6-sulfonic acid) diammonium salt) radicals according to Jarošová et al. [31]. DPPH stock solution was prepared by dissolving 25 mg of solid DPPH radical in 100 mL of methanol. The working solution was prepared from a stock solution by diluting it using 80% methanol to reach an absorbance of 0.800 ± 0.01 (λ = 515 nm) measured by GENESYS 180 Double Beam UV-Vis spectrophotometer (Thermo Fisher Scientific, Waltham, MA, USA). The reaction mixture for analysis was prepared by mixing 25 µL of the wine sample with 975 µL of the DPPH working solution. The working solution for the ABTS assay was prepared with 54.8 mg of solid ABTS radical and 1 g of MnO_2_ in 20 mL of deionized water. The solution was then filtered using PTFE (0.25 μm) syringe filters and diluted in 5 mM phosphate buffer to reach an absorbance of 0.800 ± 0.01 (λ = 734 nm), determined by GENESYS 180 Double Beam UV-Vis spectrophotometer (Thermo Fisher Scientific, Waltham, MA, USA). For the reaction mixture, 1 mL of the radical solution was mixed with 100 μL of the wine sample. L-ascorbic acid (vitamin C) was used as the reference standard for DPPH and ABTS antioxidant assays and to prepare a calibration curve in a concentration range from 0 to 2000 µg/mL. The results of both antioxidant assays were expressed as ascorbic acid equivalent in mg per 1 mL of wine sample (mg AAE/mL). The scavenging activities (%) of DPPH and ABTS radicals to compare the results with other literature were calculated by the following formula:

$$RSA=\left(\frac{Ac-As}{Ac}\right)\times 100$$

Here, *RSA* represents radical scavenging activity (%), *Ac* is the absorbance of the working solution of radical measured at 515 nm (DPPH) or 734 nm (ABTS), and *As* is the absorbance of the sample measured at 515 nm (DPPH) or 734 nm (ABTS).

### 2.8. Data Analyses

The obtained data were statistically evaluated using the Statistica 14 software program (Tibco, Palo Alto, CA, USA). To determine and compare the differences between data groups, i.e., between individual yeast strains and fruit types, or the effect of their interaction, the multi-factor analysis of variance (ANOVA) method was used. The post hoc Tukey’s HSD test was used for multiple comparisons of individual samples. Dunnett’s test was used to compare the significance of increases in pH values and antioxidant potential parameters between zero sampling (fruit juice) and the final product (pasteurized wine). Determining mutual relationships between the partially evaluated parameters was performed via Pearson correlation coefficient “r” and principal component analysis, and drawing of related plots was performed using software OriginPro 2024b (OriginLab Corporation, Northampton, MA, USA). Unless otherwise stated, differences between the tested samples were considered significant in all static evaluations at a *p*-value < 0.05 (*α* = 0.05). All correlations stated in the manuscript’s text were statistically significant (*p* < 0.05) or at a lower significance level reported in the corresponding figures.

## 3. Results and Discussion

### 3.1. Physicochemcial Properties

#### 3.1.1. Color Evaluation

Color parameters of fruit wines result from extensive biochemical transformations that fruit pigments undergo during the winemaking process, aging, and storage conditions, leading to changes in chemical composition, including in the concentration of pigments. Crucial color-forming compounds, mainly anthocyanins and carotenoids, are exposed to structural modifications during fermentation caused by physical, chemical, and microbiological factors while the chemical ones are the most relevant in the case of fruit wines [32]. The anthocyanins in the form of flavylium ions are responsible for the intense red color of fruit wines in acidic conditions. Conversely, an alkaline pH makes them unstable and causes their decomposition into dark-brown oxidized components. At slightly acidic or neutral conditions, anthocyanins interact with water, forming the unstable, colorless pseudo-bases [33]. Fruit wine color can be further affected by oxidation leading to the wine browning in the absence of protecting gas (SO_2_ or CO_2_), co-pigmentation caused by non-covalent interactions between pigments and co-pigmented molecules (non-anthocyanine phenolics like flavonols) leading to form stable polymeric pigments stabilizing wine color, or chemical changes represented by changes in pigments via hydroxylation, methylation, polymerization, and cleavage reactions [32].

All color parameters (L*, a*, and b*) of the wines differed significantly (*p* < 0.05) between the factors of the used fruit, yeast, and the sampling and their combination. The color type was determined mainly by the used fruit. While the colors of plum and hawthorn wines were reddish-orange and quite similar, the color of apple wines was in the spectrum of yellow (Figure 1). However, the differences in detailed color profiles were also obvious between wines fermented by the yeast Fermihill Fruit Red (Fruit Red) and wines fermented by the yeasts Fermihill Buket (Buket) and Fermihill Special (Special), which were similar. Moreover, different sampling times were significant from the point of color in most wine types and color parameters, as shown in Table S1. The increase in lightness (Table S1) and change in the color intensity (Figure 1) were also observed at the end of the fermentation. The same effects were reported by [34]. The authors suggest that the loss of grape wine color intensity was affected by an ethanol content higher than 6% (*v*/*v*) since this amount of ethanol can negatively affect the co-pigmentation phenomena, responsible for hyperchromatic (more intense color) and deep color shifts (purple hue) in fruit wine [32]. Anthocyanins were found in the peels of hawthorn berries [35], apples [36], and plums [37], and the inhibition of co-pigmentation by ethanol increased, leading to wine bleaching, which possibly explains the observed color differences between the fruit musts and final wines.

The obtained results showed the predominant influence of fruit type on wine color. However, the color differences were visible between yeasts and samplings. The yeasts Buket and Special were characterized by a similar color pattern within all types of fruit wine while Fruit Red differed slightly from them (Figure 1). The ethanol content also caused a decrease in wine lightness (L*).

#### 3.1.2. Changes in pH

**Figure 2:**
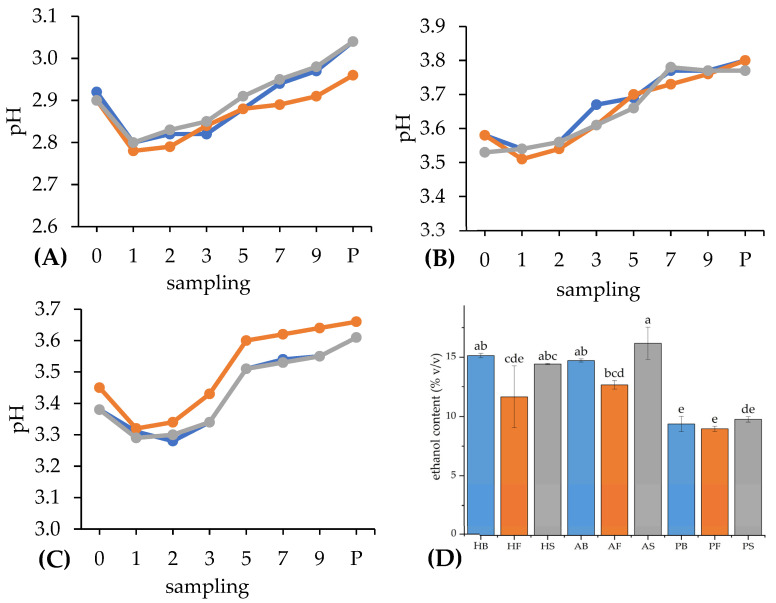
Development of pH values of hawthorn (**A**), apple (**B**), and plum (**C**) wines fermented by different yeast strains during fermentation and after pasteurization and ethanol contents of wines after pasteurization (**D**). (**A**–**C**): sampling numbers represent days of sampling and letter “P” means sampling after pasteurization; blue line: yeast “Buket”, orange line: yeast “Fruit Red”, gray line: yeast “Special”; HB: hawthorn wine fermented by yeast “Buket”, HF: hawthorn wine fermented by yeast “Fruit Red”, HS: hawthorn wine fermented by yeast “Special”, AB: apple wine fermented by yeast “Buket”, AF: apple wine fermented by yeast “Fruit Red”, AS: apple wine fermented by yeast “Special”, PB: plum wine fermented by yeast “Buket”, PF: plum wine fermented by yeast “Fruit Red”, PS: plum wine fermented by yeast “Special”. (**D**): bars represent mean of ethanol content ± standard deviation (*n* = 3) for each variant of wine and two-tail whiskers express ± standard deviation; ^a–e^ values with different superscripts within a column and the same yeast differed significantly (*p* < 0.05) based on Tukey’s HSD test.

The pH values of all fruit wines were measured in the range of 2.8–3.8 (Table S2), which nearly corresponded to the typical values of grape wines (pH 3.0–4.0), as stated in Morata et al. [38]. On the first day of fermentation, all types of fruit wines showed a slight decrease in pH. From the first to the second/third day, the trend of pH value went aside, and after the third day, a continuous increase in pH was observed on average in hawthorn and plum wines until the end of fermentation. However, in the case of plum wines, the increase in pH from the fifth to seventh day was only very slight (Figure 1. Using Dunnett’s test, a statistically significant increase in pH value (*p* < 0.05) was found for all types of fruit wines between the zero sampling day (control) and the final product in the form of pasteurized wine. For each variant of fruit wine, except the apple wine fermented by the Special yeast strain, a slight but statistically significant (*p* < 0.05) increase in pH value due to pasteurization was also found (Figure 2 and Table S2). This minor change could have been possibly caused by a slight increase in total soluble solids due to the pasteurization process, as reported by Nguyen et al. [39]. Generally, total soluble solids in fruit wines are proportional to the sugar content, forming typically neutral solutions, close to pH 7 [40].

The initial decrease in pH was potentially influenced by the disruption of the cell walls of plant tissues by naturally produced and supplemented pectinases, associated with the release of organic acids. This observation was in accordance with the study by Choi et al. [41], describing the initial pH decrease of apricot wines after the first day of fermentation, but following a pH increase after the first day. Moreover, the authors also reported lower pH values in wines treated with pectinase than in untreated control wines. The phenomenon of a decrease in pH followed by its increase in later phases of fermentation was also observed within the study by Lu et al. [42] during durian wine fermentation. The authors reported that the higher pH positively affected ethanol production while lower pH values resulted in lower ethanol production. They also mentioned the effects of fermentation temperature and pH on changes in the chemical components of durian wine. The other reason was suggested in the study by Akin et al. [43], describing the pH decrease in grape wine, which was assumed to be correlated with nitrogen consumption. During the initial fermentation phase, the consumption of nitrogen by yeasts leads to the production of H+ ions, which are responsible for the acidic pH of the solution. The study also reported a subsequent increase in pH after 40 h of fermentation, probably caused by an increasing ethanol content.

In our study, the factors of the fruit, the yeast strain, and the sampling term and the combination of these factors had a statistically significant effect (*p* < 0.05) on the measured pH values. According to the previously mentioned studies and our results, it can be suggested that various factors, including the properties of input resources and winemaking technology, may affect the dynamics of pH changes during the fermentation of fruit wines.

#### 3.1.3. Ethanol Content

The results of pycnometry analysis and statistical evaluation showed that the ethanol contents of fruit wines can be significantly affected by their origin and yeast strain. The Fruit Red yeast produced wine with the lowest ethanol of all wine types. With an average value of 11.1% ethanol, it remained deep behind the alcohol tolerance of the yeast declared by the manufacturer (15.0%). On the other hand, the yeast Special was responsible for the highest ethanol production (Figure 2D) with an average value of 13.5%, also below the value of alcohol tolerance (16.0%). The strain Buket produced wines with a slightly lower content of ethanol on average (13.1%) compared to Special. It was also a lower value than the stated alcohol tolerance (15.5%). The highest ethanol content was recorded in apple wine fermented by the Special yeast strain (16.2%), followed by hawthorn wine with the Buket strain (15.1%) and apple wine with Buket (14.4%). Using multi-factor ANOVA, we found statistically significant differences in the ethanol content between wine samples fermented by different yeasts (*p* < 0.05) and also the ones produced from different fruits (*p* < 0.05). On the other hand, the combination of these two factors had a statistically insignificant effect on ethanol content (*p* > 0.05). Tukey’s HSD multiple-comparison test (Figure 2D) showed that plum wines contained statistically lower ethanol than hawthorn and apple wines (*p* < 0.05) since the variant fermented by Fruit Red exhibited only 9.0% of ethanol. Apple wines had a higher overall ethanol percentage than plum and hawthorn wines. However, between these apple and hawthorn wines, differences were not significant (*p* > 0.05). The significantly lower ethanol content in the plum wines could be attributed to adding sucrose to the hawthorn and apple must prior to fermentation. As a disaccharide, sucrose provides a higher theoretical yield of ethanol than the monosaccharides glucose and fructose [44]. In addition to that, a combination of added sucrose with naturally occurring glucose and fructose in the case of hawthorn and apple wines can potentially cause more effective carbon utilization by yeasts, leading to their better growth and ethanol production.

According to the results, the ethanol content was affected mainly by the type of wine and to a minor extent by the yeast strain. While similar values were assessed in the case of hawthorn and apple wines on average, much lower values were observed within plum wines. From the point of view of yeast strains, wines fermented by the strain Fruit Red exhibited a lower ethanol content among all types of wine.

### 3.2. Total Polyphenol Content and Antioxidant Activity

Using multi-factor analysis of variance, statistically significant differences (*p* < 0.05) in the TPC, DPPH, and ABTS values were found between the wine types, fermentation times, and yeasts. So, each of these factors, including their interaction, affected the values of the antioxidant potential parameters. Only the sole effect of the yeast and the combination of yeast and wine type on the DPPH parameter was insignificant (Table S3). Despite the proven effect of yeast on the antioxidant potential parameters, in most cases, no clear trends were observed in developing these parameters during fermentation concerning the type of wine, the used yeast strain, and the sampling time (Figures S2–S4). Conversely, as stated in Table 1, differences between wines based on the used fruit were observed almost within every sampling, showing the average statistically highest antioxidant potential of hawthorn wines, followed by plum wines and apple wines with substantially lower TPC contents and antioxidant activities compared to hawthorn and apple wines.

As suggested by Pereira et al. [45] and Paixão et al. [46], the type of yeast, different grape varieties, and winemaking techniques, but also the geographical location or weather conditions of the site where the fruit is grown, can affect the content of polyphenols in the resulting wine and thus further affect its antioxidant activities. The ambiguous trends of the development of the TPC and related antioxidant activity during fermentation can be caused by many factors, including extraction from the fruit’s solid parts after their breakdown, especially during the initial phases of fermentation [47]. However, this phenomenon was not observed in our study, where the TPC and antioxidant activity went aside or decreased slightly. The presence of antioxidants and their activity often fluctuates during later stages of fermentation because wine is a very complex and dynamically changing matrix. As reported by two studies, the presence of polyphenols in wines decreased after their initial increase, accounting for the various chemical reactions, such as enzymatic oxidation, polymerization, and the pigmentation of phenolic compounds [48,49]. Specifically, the levels of anthocyanins, which are the dominant antioxidant pigments of plums, apples, and hawthorn, as stated in Section 3.1.1, may decrease due to the adsorption on the solid particles in wine, degradation reactions, structural changes, and the formation of polymeric pigments with tannins [47]. Hensen et al. [50] reported that pectin may form complex structures with anthocyanins and tannins. Pectins can reduce the concentration of tannins by forming insoluble complexes. Another study described the decomposition of pectin during the fermentation and further complexation of rhamnogalacturonan II with anthocyanins [51]. These reactions do not only affect the sensory parameters of wine like color and astringency, as these studies described, but also may ambiguously affect the extraction of polyphenols during fermentation, as shown in the description of these effects within grape wines [52]. In this study, pectic polysaccharides protected monomeric flavanols and tannins in Pinot noir, thereby increasing their concentrations. On the other hand, polysaccharides precipitated or masked these compounds in Cabernet Sauvignon, thus reducing total polyphenol content. These studies may have justified the discrepancy in TPC dynamics during fermentation between apple and other wines. Also, the other phenolic compounds may undergo changes that lead to the loss of antioxidant activity. Razmkhab et al. [53] reported that flavanol derivatives and colored products formed from phenolic oxidation or condensation reactions may be retained by both yeasts and their cell walls. These findings suggest that the polyphenol content and its antioxidant activity may be affected during fermentation by many factors, including extraction, structural changes, and various chemical or physical reactions. Therefore, without studying these phenomena on the molecular level, it is impossible to elucidate the ambiguous development of the TPC and antioxidant activity of the fruit wines produced in our study.

Using Dunnett’s test, statistically significant differences were found (*p* < 0.05) between the zero sampling day (control) and the final product in the form of pasteurized wine, regardless of the used yeast, in the case of hawthorn wine and the DPPH parameter (higher value compared to the control) and within apple wine for the TPC parameter (lower value compared to the control), DPPH (higher value compared to the control), and ABTS (higher value compared to the control).

An unambiguous trend of a decreasing TPC during the whole fermentation process was observed only for apple wines (Figure S2), which was in contrast to their previously mentioned increase in antioxidant activity. Nevertheless, these results were in accordance with the study by Tarko et al. [54], which also observed a decrease in the TPC during apple wine production but a slight increase in antioxidant activity determined by ABTS assay. Our results regarding the TPC and antioxidant assays were similar to the results described in the study by Rupasinghe and Clegg [55], which reported TPC values as 0.40 mg GAE/mL determined by Folin–Ciocâlteu method and 0.45 mg AAE/mL assessed by the FRAP method for unpasteurized apple wine.

The results of the TPC in hawthorn wines exhibited lower values ranging between 1.9 and 2.4 GAE/mL (Table 1) compared to the 3.1–3.5 mg GAE/mL reported by He et al. [56], which also produced hawthorn (*Crataegus pinnatifida*) wine by different wine yeasts. This study also reported an above-60% scavenging activity of radical DPPH, a more-than-79–84% scavenging activity of the superoxide anion radical, and a high capability of hawthorn wines to scavenge ABTS radicals and ferric-reducing antioxidant power. The authors found a strong correlation between the TPC and antioxidant assays and significant differences in the antioxidant potentials of hawthorn wines produced by various wine yeasts. Since the scavenging activities against the DPPH and ABTS radicals of hawthorn unpasteurized wines produced within our study were lower (Table 1), specifically for DPPH in a range of 33–38% and for ABTS between 49–54%, this should be considered a different approach in the preparation of original hawthorn must before fermentation, where, in our study, the hawthorn berries were mixed with water in a ratio of 1:3 but in the cited manuscript, the ratio was 1:1.5. Another study reported the TPC contents of differently thermally, microwave-treated, and untreated hawthorn wines between 1.7 and 2.1 mg GAE/mL, which were lower compared to our values [57]. An older study by the authors reported lower TPC values (1.0–1.1 mg GAE/mL) for a fermented hawthorn drink [58].

Some studies focusing on determining the TPC and antioxidant scavenging activities of plum wines were published. One of the studies reported approximately 55% scavenging activity for the DPPH radical, which was higher than the 40% scavenging activity of plum wines from our study (Table 1). However, the ABTS scavenging activity (32%) was lower than our values (38%) [59]. Another study, on the other hand, reported 40% scavenging activity against DPPH in wines from Prunus salicina Lindl., which was the same as our result [60]. This study also reported that the TPC content reached 1.6 mg GAE/mL, the same value as that found in our pasteurized plum wines. The TPC of unpasteurized wines within our study was slightly lower (1.5 mg GAE/mL). The other study reported the TPC of plum wines prepared from three different cultivars of *Prunus domestica* L. These wines exhibited TPC contents in the range of 1.7–2.2, showing the variability of plum cultivars, confirming that different genotypes of various fruits, also, significantly affect the phenolic contents and related antioxidant potentials of produced wines [61].

By using a multi-factor analysis of variance when comparing the antioxidant potential parameters of samples before and after pasteurization, it was found that the pasteurization of fruit wines in general did not significantly affect the change in TPC values. These findings could be supported by a study from Nguyen et al. [39], who also did not observe significant changes in the TPC of beetroot wine after pasteurization. As was reported in the other study, polyphenols tend to be relatively resistant to thermal processing since the highest yield of polyphenols during extraction is reached at 60–80 °C [62], so a gentle pasteurization process, like the one used in this study, should not cause their degradation or deactivation, which was coherent with our data (Table 1). Moreover, it was shown that pasteurization caused an increase in the values of TPC, except in the apple wines.

Statistical evaluation proved a significant (*p* < 0.05) effect of pasteurization within DPPH and ABTS antioxidant assays. As Table 1 indicates, in most cases, when averaging the values for all yeasts, the parameter values increased after pasteurization, except for the values of DPPH and ABTS in plum wines. However, the decrease in antioxidant activity at plum wines was minimal. On the contrary, for some samples (hawthorn DPPH, hawthorn ABTS), the average increase in values was significant (*p* < 0.05). Like for the TPC, it can be said that pasteurization with the parameters used in this work generally does not have an unambiguous effect on the changes in antioxidant potential parameters, especially due to the variability in the inner factors of the used material like the chemical composition of the used fruit and thermal-derived changes in bioactive compounds. Nevertheless, some specific cases showed the potential of the pasteurization process to increase the antioxidant activity of fruit wines significantly. This observation corresponded to the study by Ferreira et al. [63], in which the authors found that fermented beverages from opuntia showed increased antioxidant activity compared to the original fruit juice before fermentation using the ABTS and DPPH methods. The authors also confirmed that pasteurization does not necessarily reduce the biological value of fruit wines and may even slightly increase it in some cases, which implies that more research in this field is needed.

Based on the obtained results, it can be concluded that although the influence of all observed factors on the results of the antioxidant potential parameters was confirmed, these properties are predominantly determined by the chemical composition of the used fruit since it was not possible to observe a general trend of change in these parameters during fermentation depending on the applied yeast strain. Several studies have reported confirming the effects of the fermentation of fruit wines by *Saccharomyces*, non-Saccharomyces, and mixed cultures on the TPC and antioxidant activities of fruit wines. However, also, these studies have not confirmed the unambiguous effect of the specific yeast strains on these parameters [2]. To select the particular yeasts potentially capable of improving the biological activities of the original fruit juices via fermentation and utilize this phenomenon in winemaking practice, it would be necessary to perform a targeted study including a wide spectrum of commercial *Saccharomyces* wine yeast strains or the applicable wild-type strains.

### 3.3. Sensory Analyses

**Figure 3:**
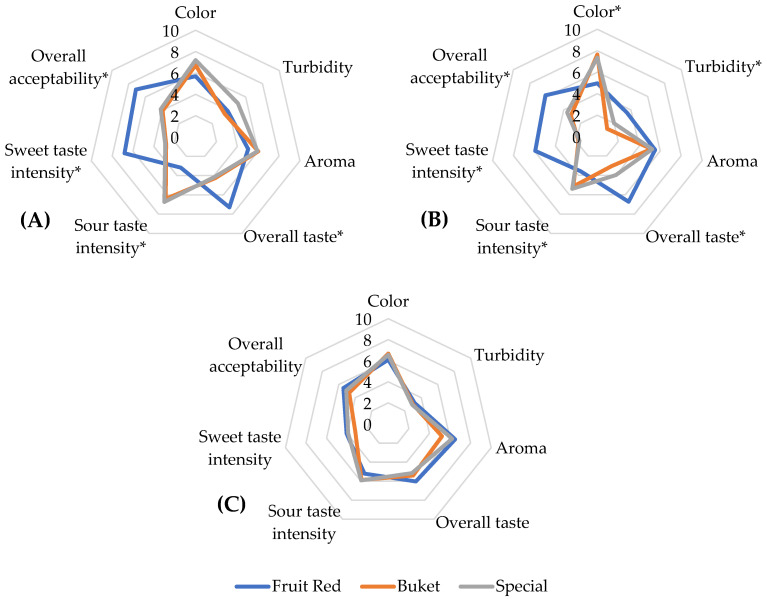
Sensory analysis (0 = unpleasant, 10 = very pleasant) of hawthorn (**A**), apple (**B**), and plum wines (**C**) fermented by yeast strains Buket, Fruit Red, and Special after pasteurization. Results are presented as means (n = 26); *: statistically significant difference (*p* < 0.05) between yeast strains within descriptor.

Statistical evaluation confirmed significant differences (*p* < 0.05) in almost all sensory descriptors between wine types and yeasts, including the interaction of both factors, except for the descriptor of aroma (Table S4 and Figure 3). Statistical insignificances were also observed in color between individual wine types and differences in turbidity when combining the wine type and yeast (Table S5). In general, these results suggest that the fruit and selection of yeast species and its strain play a key role in shaping the sensory properties of fruit wines. Specifically, the yeast strain Fruit Red had a significant positive effect on the overall taste and overall acceptance descriptors, which was strongly reflected in the sensory profiles shown in Figure 3.

The differences in the sensory properties of wines fermented with different yeasts confirm the preference for wines fermented with the *S. cerevisiae* strain over those fermented with the *S. bayanus* strain. This observation was also reported in a study focused on the sensory properties of plum wines, where the must fermented with *S. cerevisiae* strains had a better taste and overall impression than wines fermented with *S. bayanus* yeast [64]. This result was consistent with our findings, where wines fermented by Fruit Red (*S. cerevisiae*) were evaluated better regarding taste and overall acceptance than those made with Buket and Special, representing strains of *S. bayanus*. As shown in Figure 3C, the differences in descriptors within three plum wines fermented by various yeasts were irrelevant. However, the radar charts of the sensory evaluation of apple and hawthorn wines (Figure 3) show the clearly different sensory profiles of wines fermented by Fruit Red than those fermented by the Buket and Special yeasts. Moreover, the total sensory profiles of wines of these two strains were very similar.

Surprisingly, low and statistically insignificant (*p* > 0.05) differences in aroma pleasantness were found. This parameter was evaluated similarly in the narrow scale of 5.0–6.2 points within all wine types. It could indicate the low sensitivity of panelists to favor certain types of wine or yeast over others in their evaluation of aroma pleasantness. In a study by Coelho et al. [65], trained panelists also performed sensory analyses of orange, mango, cherry, and banana wines. However, these evaluators could clearly distinguish the aromatic profiles of the wines and determine a characteristic olfactory descriptor for each. It can be assumed that plum, apple, and hawthorn wines had comparable aromatic intensity values, possibly less distinguishable than the aroma attributes of the fruits used in the mentioned study. Despite this result, it does not necessarily mean no aromatic differences existed between the wines since the single wine samples were rated in the range of unacceptable to excellent aromas by individual panelists in some cases. Moreover, sensory analysis only evaluated the general pleasantness of the aroma, not its specific character (e.g., fruity, floral, or other tones). These nuances could be reliably evaluated only by professional panelists. Although the evaluators may have perceived differences in aroma between the samples, the absence of more detailed descriptors often tended them to classify all wines in the same category of pleasantness, typically in the middle position on the hedonic scale.

When comparing the ethanol content with the sensory evaluation of fruit wines, it was found that the apple wines with a higher ethanol content (fermented by the Buket and Special yeasts) achieved worse sensory results than the wine with a lower ethanol content with the Fruit Red yeast. The same result was observed in the case of hawthorn wines. According to Zhang et al. [66], who conducted a comprehensive study of the chemical and sensory profiles of hawthorn wines from China, an increasing ethanol concentration can suppress the perception of sweet aromas and flavors, which shows the close relationships between the sensory properties and ethanol contents of analyzed wines.

The sensory analysis results confirmed the significant importance of yeast selection for fruit wine production as shown especially in the case of apple and hawthorn wines in this study. While wines made from the Buket and Special yeast had similar sensory profiles, wines made with the Fruit Red yeast showed significantly better parameters in overall taste, sweet taste intensity, and overall acceptability, which strongly correlated, as discussed in more detail in Section 3.4. This yeast strain also provided the best parameters for plum wine although they were not statistically significant (*p* > 0.05) compared to the parameters obtained using other yeasts. Panelists from the Czech Republic rated the hawthorn wine produced using the Fruit Red yeast as the best of all the wines evaluated within our study from the point of overall taste and overall acceptance (Table S5). This result is especially valuable in the context of Chinese hawthorn, which is not a commonly occurring fruit species in the Czech Republic or Europe and is, therefore, not a common raw material for fruit wine production in that region. Moreover, the rather bitter–sour or tart taste and relatively hard texture of hawthorn, combined with its low juice content, high pectin contents, and related difficult processing, are not parameters that would directly predetermine the use of this fruit for wine production. Therefore, the results confirm that choosing a suitable wine yeast strain can substantially influence the sensory properties of wines produced from less traditional or non-typical fruits for winemaking.

Our results showed that wines fermented by the strain Fruit Red exhibited the highest sweet taste intensity perception and best overall taste and acceptance, showing the unambiguous effect of the yeast strain on fruit wines’ sensory properties. Moreover, the wines fermented by Fruit Red provided substantially different sensory profiles in hawthorn and apple wines than those of the fruits fermented by the strains Buket and Special. On the other hand, the sensory profiles of plum wines were similar, so the effect of yeast strain was insignificant compared to that of wines obtained from other fruits.

### 3.4. The Relationships Between Observed Properties

**Figure 4:**
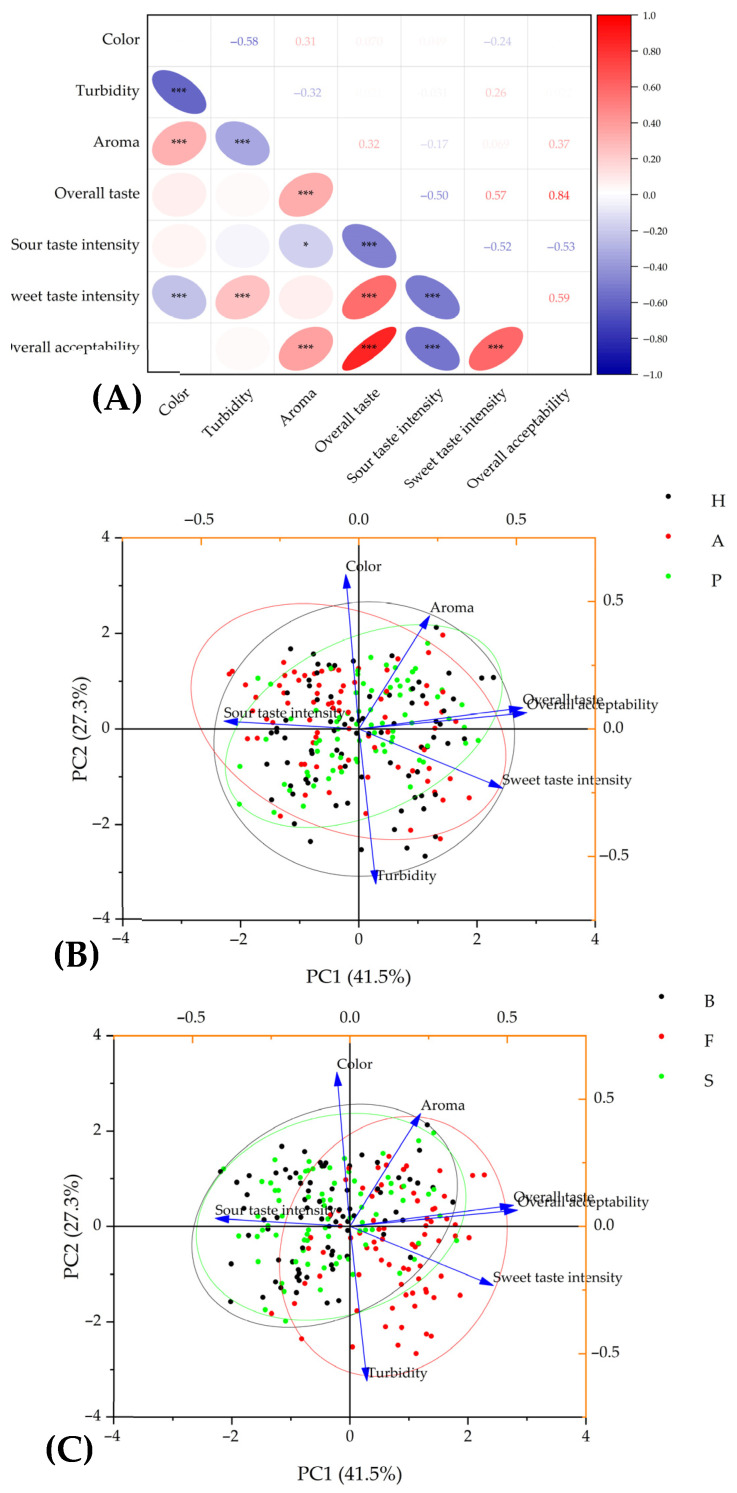
Correlation analysis (**A**) and principal component analysis biplot of sensory parameters of hawthorn, apple, and fruit wines fermented by different fruits (**B**) and yeasts (**C**). Correlation values are shown in the upper triangular; significant marks shown in the lower triangular; *, and *** show significant difference at the 0.05 and 0.001 probability levels, respectively. H: hawthorn wines, A: apple wines, and P: plum wines; B: yeast “Buket”; F: yeast “Fruit Red”; S: yeast “Special”.

Based on the correlation analysis of sensory descriptors (Figure 4A), a strong positive relationship between overall taste and overall acceptability (r = 0.84) confirmed that taste plays a key role in consumer preference. At the same time, a moderately strong positive correlation was found between the intensity of sweet taste intensity and overall taste (r = 0.57) and overall acceptability (r = 0.59), from which it can be concluded that the panelists positively appreciated a sweet taste over a sour taste. Conversely, a sour taste was significantly negatively correlated with all three descriptors (Figure 4). These findings were consistent with a study by Zhu et al. [67], according to which consumers generally preferred fruity wines with sweetness, moderate acidity, and a balanced aroma, which they reported with the example of blueberry wine. On the contrary, wines with excessive acidity, bitterness, or astringency were rated negatively and considered less attractive in this study. The observed phenomenon of the strong correlation of sweetness with overall taste and acceptability within our study can be explained by the sensory panel composition. As observed in the study by Sena-Esteves et al. [68], females and novices tended to prefer the sweeter samples of red wines compared to men and experienced evaluators. In our groups, men were in the minority, and experienced consumers were neither involved, possibly explaining the strong preference of the samples with high sweet taste intensity. Acidity and sweetness have a well-documented balancing relationship. Ailer et al. [69] reported that higher acidity can make a wine with sugar taste less sweet. However, this interaction is crucial for the overall harmony of the wine. Compared to apple and hawthorn wines fermented by the yeast strains Buket and Special, these wines fermented by Fruit Red were characterized by a lower intensity of sour taste and, on the other hand, by a higher intensity of sweet taste. The acidity of wines is mainly determined by the content of organic acids occurring in raw materials or as the products of yeasts during the winemaking process. Scutarașu et al. [70] focused on the occurrence and production of various organic acids during fermentation and the sensory profiles of final samples of white wines, which were crucially affected by the applied yeast strain. This observation may explain and justify such a big influence of yeast strain on the sensory profile of hawthorn, apple, and, to a minor extent, plum wines. We also observed that turbidity negatively affected the perception of color (r = −0.58), highlighting the importance of wine clarity important for the acceptance of a wine.

Using the PCA analyses, two component areas, PC1 (41.5%) and PC2 (27.3%), were distinguished, accounting for 68.8% of the total data variance (Figure 4B,C). The first PCA plot (Figure 4B) suggests the ambiguous effect of different fruits on the sensory parameters since the scores of the single wines regarding fruits are not separated. Therefore, these three clusters overlap strongly. The second PCA plot (Figure 4C) shows the similarity of sensory parameters determined by the yeast strains Buket (black cluster) and Special (green cluster), inclining more towards the sour taste sensory parameter variable. On the other hand, the red cluster representing the yeast strain Fruit Red exhibits smaller overlap with the two other clusters and is more associated with other variables representing overall acceptability, overall taste, and sweet taste intensity, which correlate together, as mentioned previously. These data support the advantage of using the Fruit Red yeast to produce fruit wines from the point of sensory properties compared to the two other yeast strains.

**Figure 5:**
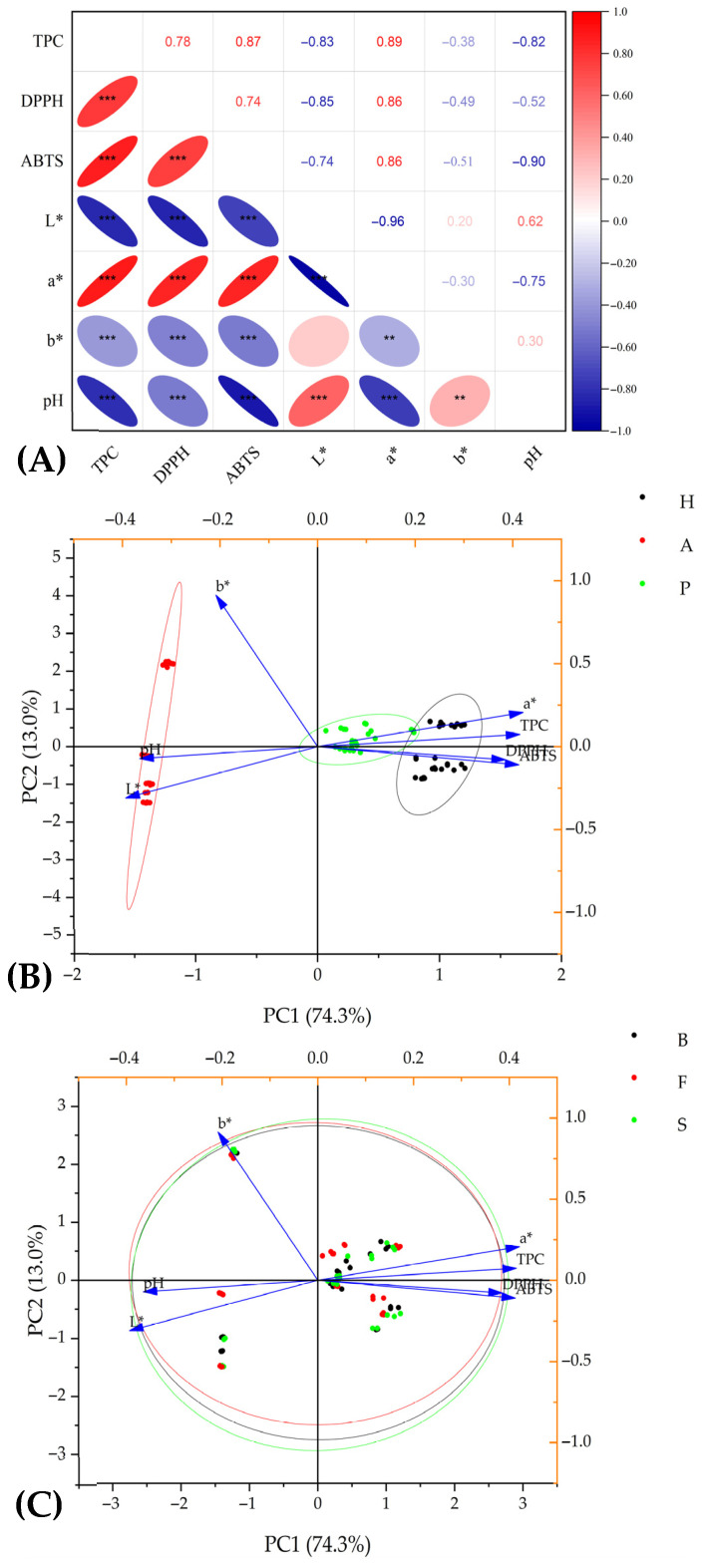
Correlation analysis (**A**) and principal component analysis biplot of physicochemical and antioxidant potential parameters of hawthorn, apple, and fruit wines fermented by different fruits (**B**) and yeasts (**C**). Correlation values are shown in the upper triangular; significant marks shown in the lower triangular; ** and *** show significant difference at the 0.01, and 0.001 probability levels, respectively. TPC: total polyphenol content; DPPH: DPPH antioxidant assay; ABTS: ABTS antioxidant assay; L*, a*, b*: parameters of CIELAB color space; H: hawthorn wines; A: apple wines; P: plum wines; B: yeast “Buket”; F: yeast “Fruit Red”; S: yeast “Special”.

The correlation analysis of antioxidant potential parameters and physicochemical properties showed both expected and interesting findings (Figure 5). As expected, strong positive correlations were found between the individual antioxidant potential parameters, which was coherent with the study by Paixão et al. [27]. In the cited study, the TPC was strongly positively correlated with antioxidant activities determined by DPPH, ABTS, and FRAP assays within the various types of grape wines. Another study reported a significant positive correlation of the TPC with FRAP but also a strong negative correlation with DPPH in strawberry and drupe (plum, peach, apricot, and sweet cherry) wines [71].

This negative correlation between the TPC and DPPH can be affected by possibly distorted results of DPPH assay due to acidic pH conditions, as stated in the research by Ferri et al. [72]. The authors reported that DPPH is unstable and gives false-positive results and therefore recommend a pH range between 4 and 8 for this assay. This finding can indicate why all antioxidant potential parameters correlated negatively with the pH of wines ranging from 2.8 to 3.8. Moreover, it can potentially explain the lower values of DPPH scavenging activities of hawthorn wines while their values of TPC and scavenging activity against ABTS were significantly higher compared to plum wines (Table 1).

Interestingly, the values of the parameters of antioxidant potential were closely related to all parameters of the L*a*b* color space. The L* value defining the lightness of the sample was strongly negatively correlated with the TPC, DPPH, and ABTS values (Figure 5), suggesting that the darkness or intensity of wine color can be proportionally related to the content of colored components with antioxidant activity. Conversely, these parameters correlated positively with the a* value, which defines the range from green (low negative values) to red (high positive values). These findings were reported by Pereira et al. [45] or Paixão et al. [46], according to which phenolic compound concentrations are generally higher in red wines than in white wines. On the other hand, the negative correlation with the b* parameter, which ranges from blue (low negative values) to yellow (high positive values), may indicate that fruit juices or wines that are yellow or have a pale color can be associated with lower antioxidant potential. This hypothesis is supported by the fact that yellowish apple wines (Figure 1) had significantly lower antioxidant potential parameters than reddish-orange colored wines made from hawthorn and plums, as shown in Table 1.

Similar findings were reported by Yildirim [73], who appointed the highest antioxidant potential of the bilberry, blackberry, and black mulberry wines, which also belonged to the wines with the highest color parameters. In this study, total phenolics and antioxidant activities also correlated significantly, and both parameters correlated with most of the determined color-associated parameters. The mentioned fruit types are rich sources of anthocyanins and other polyphenols [74]. These results suggest that dark-colored (like deep red, blue, or black) fruits and wines produced from them usually possess strong antioxidant activity.

PCA biplots based on PC1 and PC2 showed 87.3% of total data variance, illustrating the strong effect and variability of different wines on the selected physicochemical and antioxidant potential (Figure 5B,C). Three, clearly separated clusters indicated the big differences between fruit wines based on their origins and the fruits used for their production (Figure 5B). The red cluster, representing the apple wines, was associated with higher pH and L* and b* color components. The other two clusters, representing plum (green) and hawthorn (black) wines, were quite distant from the red cluster and were associated with antioxidant potential and a* color component variables. The scores representing hawthorn wine samples were closest to the mentioned variables, confirming the highest antioxidant potential of this type of wine from all three types of fruit wine, regardless of the used yeast strain. On the other hand, the second PCA plot (Figure 5C) showed the nearly perfectly overlapping circle outlines of yeast-related clusters, suggesting the negligible effect of yeast strain on certain antioxidant and physicochemical parameters, compared to the effect of the fruit type on these traits.

The correlation and PCA analysis revealed interesting relationships between determined parameters. The sensory acceptance of evaluated fruit wines was closely positively related to the sweetness of a wine, which also affected the overall taste. On the other hand, a higher intensity of sources was negatively reflected in overall taste and acceptance. As expected, a better evaluation of aroma also positively and significantly correlated with overall taste and acceptance. Analysis of the relationship between physicochemical properties and antioxidant potential parameters showed the close relation of wine color and the TPC and related antioxidant activities. The values of color components related to the dark, red, and blue (opposite to light, green, and yellow) colors were very strongly positively correlated with the TPC and antioxidant activity parameters. Correspondingly, darker and reddish-orange colored hawthorn and plum wines had significantly higher TPCs and antioxidant properties than yellowish apple wines. The TPC and antioxidant parameters were very strongly and positively correlated, and these parameters were negatively correlated with pH values.

## 4. Conclusions

This study proved the significant impact of wine yeast on the physicochemical, sensory and antioxidant parameters of wines made from plum, apple, and hawthorn fruits. Three selected winery yeast strains affected the dynamics of pH changes, ethanol content, and color attributes differently. It was also shown that the mild pasteurization of fruit wines mostly did not decrease their qualitative parameters or biological values. The TPC and antioxidant content usually remained unchanged or increased. Despite this, the TPC of apple wines on average decreased after pasteurization, and a slight decrease in antioxidant activities was observed in most of the plum wines. These results generally suggest the potential usability for preserving these traits and increasing shelf life simultaneously. However, some cases of negative effects on antioxidant potential indicate that further research in this area is needed. The yeast strain also significantly affected the TPC and antioxidant activities during fermentation. However, the trends of their values were ambiguous, indicating that the chemical composition of raw fruits used for winemaking remained the most important factor determining the biological potential of the obtained wines. On the other hand, the significantly positive effect of yeast selection in forming the sensory profile of fruit wines was proven. The yeast Fruit Red provided the best results from the taste and overall acceptability parameters, which was especially significant in the case of apple and hawthorn wines. Hawthorn wine fermented by this yeast was evaluated as the best variant from the point of sensory analysis, confirming the potential of this rigid fruit in comparison with traditional and simply processable fruits like apples or plums. The correlation analysis confirmed the strong relation of sweetness intensity with the taste and overall acceptability of fruit wines. The total polyphenol content was strongly correlated to antioxidant activities, and simultaneously, these parameters were closely related to the colors of wines, showing that color intensity and red-colored hawthorn and plum wines had higher bioactive potential.

In practice, the complex and accessible results and found relations of this study could allow homemade or commercial fruit wine producers to navigate more simply through the potential of selected types of fruits, their processing, and the pasteurization of wines and show the various effects of yeast and the mentioned parameters during winemaking on the sensory profile and biological potential of the final products. They also suggest the wine parameters, predominantly determined by the chemical composition of fruits, which can thus hardly be influenced by yeast or the fermentation process. The robust sensory analysis approach can be useful in easily determining the preferences of fruit wine consumers based on the fruit and yeast strains used for wine production. Therefore, the study’s findings can be reflected in the important aspects of the fruit winemaking process, like in the targeted selection and processing of plant raw materials and yeast strain selection, leading to the obtainment of fruit wines with properties desirable for consumers, especially in terms of sensory parameters and health-promoting effects.

## Figures and Tables

**Table 1:** Total polyphenol content and scavenging activity against radicals DPPH and ABTS of hawthorn, apple, and plum wines fermented by different yeast strains in the zeroth and ninth days and after pasteurization.

		TPC			DPPH			ABTS		
S	Y	Hawthorn	Apple	Plum	Hawthorn	Apple	Plum	Hawthorn	Apple	Plum
0	B	2.14 ± 0.20 ^Aabc^	0.81 ± 0.02 ^Ba^	1.41 ± 0.43 ^Bb^	1.09 ± 0.06 ^Aab^	0.36 ± 0.05 ^Bb^	1.08 ± 0.27 ^Aab^	2.82 ± 0.08 ^Ab^	0.58 ± 0.02 ^Cd^	1.73 ± 0.02 ^Ba^
	F	2.44 ± 0.11 ^Aa^	0.82 ± 0.05 ^Ca^	1.86 ± 0.04 ^Bab^	1.14 ± 0.07 ^Aab^	0.33 ± 0.03 ^Cb^	0.92 ± 0.08 ^Bb^	2.97 ± 0.04 ^Aa^	0.45 ± 0.01 ^Cf^	1.61 ± 0.03 ^Bcd^
	S	2.32 ± 0.06 ^Aab^	0.77 ± 0.02 ^Ba^	2.32 ± 0.41 ^Aa^	1.06 ± 0.06 ^Aab^	0.32 ± 0.05 ^Bb^	1.22 ± 0.23 ^Aab^	2.94 ± 0.03 ^Aab^	0.53 ± 0.00 ^Ce^	1.35 ± 0.01 ^Bf^
9	B	2.02 ± 0.06 ^Abc^	0.50 ± 0.01 ^Cc^	1.45 ± 0.07 ^Bb^	1.13 ± 0.01 ^Aab^	0.41 ± 0.02 ^Bab^	1.16 ± 0.08 ^Aab^	2.41 ± 0.01 ^Ac^	0.63 ± 0.01 ^Cbc^	1.71 ± 0.08 ^Ba^
	F	2.25 ± 0.15 ^Aabc^	0.63 ± 0.03 ^Cb^	1.65 ± 0.04 ^Bb^	1.07 ± 0.13 ^Aab^	0.32 ± 0.06 ^Bb^	1.29 ± 0.07 ^Aab^	2.25 ± 0.02 ^Ad^	0.62 ± 0.01 ^Cc^	1.55 ± 0.02 ^Bde^
	S	2.14 ± 0.06 ^Aabc^	0.48 ± 0.02 ^Cc^	1.45 ± 0.02 ^Bb^	0.97 ± 0.06 ^Bb^	0.41 ± 0.03 ^Cab^	1.20 ± 0.09 ^Aab^	2.51 ± 0.05 ^Ac^	0.63 ± 0.00 ^Cbc^	1.67 ± 0.09 ^Bab^
P	B	2.36 ± 0.17 ^Aab^	0.48 ± 0.01 ^Cc^	1.67 ± 0.07 ^Bb^	1.14 ± 0.07 ^Aab^	0.43 ± 0.02 ^Bab^	1.16 ± 0.19 ^Aab^	2.93 ± 0.03 ^Aab^	0.68 ± 0.01 ^Ca^	1.52 ± 0.02 ^Be^
	F	1.91 ± 0.06 ^Ac^	0.52 ± 0.02 ^Cc^	1.57 ± 0.02 ^Bb^	1.16 ± 0.03 ^Bab^	0.34 ± 0.04 ^Cb^	1.41 ± 0.03 ^Aa^	2.87 ± 0.01 ^Aab^	0.58 ± 0.02 ^Cd^	1.64 ± 0.01 ^Bbc^
	S	2.24 ± 0.24 ^Aabc^	0.51 ± 0.03 ^Cc^	1.66 ± 0.03 ^Bb^	1.24 ± 0.06 ^Aa^	0.48 ± 0.01 ^Ca^	1.01 ± 0.13 ^Bab^	2.93 ± 0.06 ^Aab^	0.66 ± 0.01 ^Cab^	1.69 ± 0.03 ^Bab^
9 (RSA)	B	-	-	-	37.5 ± 0.2 ^Aa^	14.8 ± 0.6 ^Ba^	38.3 ± 2.6 ^Aa^	52.1 ± 0.3 ^Ab^	30.8 ± 0.3 ^Ca^	38.9 ± 0.4 ^Ba^
	F	-	-	-	35.5 ± 4.1 ^Aa^	12.2 ± 1.9 ^Ba^	42.3 ± 2.1 ^Aa^	49.1 ± 0.4 ^Ac^	30.2 ± 0.4 ^Ca^	36.1 ± 0.4 ^Bb^
	S	-	-	-	32.5 ± 1.8 ^Ba^	14.9 ± 0.9 ^Ca^	39.7 ± 2.8 ^Aa^	53.9 ± 0.9 ^Aa^	30.7 ± 0.1 ^Ca^	38.3 ± 0.3 ^Ba^

**S**: sampling; **Y**: yeast; B: Buket, F: Fruit Red, S: Special; TPC: Total polyphenol content; DPPH: 2,2-diphenyl-1-picrylhydrazyl; ABTS: [2,2-azinobis-(3-ethylbenzothiazoline −6-sulfonic acid)]; RSA: radical scavenging activity. TPC content values are expressed in mg of equivalent of gallic acid per ml of wine; scavenging activities against radicals DPPH and ABTS are expressed in mg of equivalent of ascorbic acid per ml of wine. RSA: relative radical scavenging activity expressed as % of quenched radical (DPPH or ABTS) absorbance by fruit wine. Results are presented as means ± standard deviations (*n* = 3); ^a–f^ values with different superscripts (small letters) within a column and the same parameter of antioxidant potential differed significantly (*p* < 0.05) based on Tukey’s HSD test; ^A–C^ values with different superscripts (big letters) within a row and single assay (TPC, DPPH, or ABTS) differed significantly (*p* < 0.05) based on Tukey’s HSD test.

## Data Availability

The original contributions presented in the study are included in the article, further inquiries can be directed to the corresponding author.

## Supplementary Materials

The following supporting information can be downloaded at: https://www.mdpi.com/article/10.3390/foods14162844/s1, Figure S1: Calculation table for the determination of ethanol content in fruit wines based on density; Figure S2: Total polyphenol contents of hawthorn, apple, and plum wines fermented by yeast strains Buket, Fruit Red, and Special during fermentation and after pasteurization; Figure S3: Development of scavenging activity of hawthorn, apple, and plum wines fermented by yeast strains Buket, Fruit Red, and Special during fermentation and after pasteurization against radical DPPH; Figure S4: Development of scavenging activity of hawthorn, apple, and plum wines fermented by yeast strains Buket, Fruit Red, and Special during fermentation and after pasteurization against radical ABTS; Table S1: Evaluation of color of hawthorn, apple, and plum wines fermented by different yeast strains on the zeroth and ninth days and after pasteurization using CIELAB (L*a*b*) color space; Table S2: Development of pH value of hawthorn, apple, and plum wines fermented by different yeast strains during fermentation and after pasteurization; Table S3: Statistical analysis of significant differences between fruit wine types, yeasts, and samplings and the combined effect of these factors within antioxidant potential parameters of pasteurized wines from hawthorn, apple, and plum wines fermented by different yeast strains; Table S4: Statistical analysis of significant differences between fruit wine types, yeasts, and the combined effect of fruit and yeast within sensory parameters of pasteurized wines from hawthorn, apple, and plum wines fermented by different yeast strains; Table S5: Values of the sensory parameters (0 = dislike extremely, 10 = like extremely) of hawthorn, apple, and plum wines fermented by different yeast strains after pasteurization.
