# Supplementary material for: Effect of Wine Yeast (Saccharomyces sp.) Strains on the Physicochemical, Sensory, and Antioxidant Properties of Plum, Apple, and Hawthorn Wines
Source: Foods. 2025 Aug 16;14(16):2844. doi: 10.3390/foods14162844 (PMC12386142; doi:10.3390/foods14162844)
Supplement: Supplementary file 1 [file foods-14-02844-s001.zip › foods-3792871-supplementary.pdf]

foods

Article

Supplementary material:

Effect of Wine Yeast (*Saccharomyces* sp.) Strains on the Physicochemical, Sensory, and Antioxidant Properties of Plum, Apple, and Hawthorn Wines

František Lorenc [1,*], Markéta Jarošová [2], Jan Bedrníček [1], Vlastimil Nohejl [1], Eliška Míková [1] and Pavel Smetana [1]

[1] Department of Food Biotechnologies and Agricultural Products' Quality, Faculty of Agriculture and Technology, University of South Bohemia in České Budějovice, Studentská 1668, 370 05 České Budějovice, Czech Republic; bedrnicek@fzt.jcu.cz (J.B.); nohejl@fzt.jcu.cz (V.N.); mikove00@fzt.jcu.cz (E.M.); smetana@fzt.jcu.cz (P.S.)

[2] Department of Plant Production, Faculty of Agriculture and Technology, University of South Bohemia in České Budějovice, Na Sádkách 1780, 370 05 České Budějovice, Czech Republic; jarosovam@fzt.jcu.cz (M.J.)

* Correspondence: lorencf@fzt.jcu.cz (F.L.)

foods

MDPI

h20°C/20 °C	EtOH % vol.	h 20°C/20 °C	EtOH % vol.	h 20°C/20 °C	EtOH % vol.
1,000	0,00	57	2,91	13	6,11
0,9999	0,07	55	3,05	12	6,18
98	0,13	54	3,12	11	6,26
97	0,20	53	3,19	0,9910	6,24
96	0,26	52	3,26	09	6,41
95	0,33	51	3,33	08	6,59
94	0,40			07	6,57
93	0,46	0,9950	3,40	06	6,65
92	0,53	49	3,47	05	6,73
91	0,60	48	3,54	04	6,80
		47	3,61	03	6,88
0,9990	0,66	46	3,68	02	6,96
89	0,73	45	3,76	01	7,04
88	0,80	44	3,83		
87	0,87	43	3,90	0,9900	7,12
86	0,93	42	3,97	899	7,19
85	1,00	41	4,04	98	7,27
84	1,07			97	7,35
83	1,14	0,9940	4,11	96	7,43
82	1,20	39	4,18	95	7,51
81	1,27	38	4,26	94	7,59
		37	4,33	93	7,67
0,9980	1,34	36	4,40	92	7,75
79	1,41	35	4,48	91	7,82
78	1,48	34	4,55		
77	1,54	33	4,62	0,9890	7,90
76	1,61	32	4,69	89	7,98
75	1,68	31	4,77	88	8,06
74	1,75			87	8,15
73	1,81	0,9930	4,84	86	8,23
72	1,88	29	4,91	85	8,31
71	1,95	28	4,98	84	8,39
		27	5,06	83	8,47
0,9970	2,02	26	5,13	82	8,55
69	2,09	25	5,21	81	8,63
68	2,15	24	5,58		
67	2,22	23	5,36	0,9880	8,71
66	2,29	22	5,43	79	8,79
65	2,36	21	5,51	78	8,88
64	2,43			77	8,96
63	2,50	0,9920	5,58	76	9,04
62	2,57	19	5,66	75	9,13
61	2,64	18	5,73	74	9,21
		17	5,81	73	9,29
0,9960	2,70	16	5,88	72	9,38
59	2,77	15	5,96	71	9,46
58	2,84	14	6,03		

h20°C/20 °C	EtOH % vol.	h 20°C/20 °C	EtOH % vol.	h 20°C/20 °C	EtOH % vol.
69	9,62	26	13,25	82	17,20
68	9,70	25	13,34	81	17,29
67	9,79	24	13,43		
66	9,87	22	13,60	79	17,47
65	9,95	21	13,68	78	17,57
64	10,03			77	17,66
63	10,11	0,9820	13,77	76	17,75
62	10,20	19	13,86	75	17,84
61	10,28	18	13,95	74	17,94
		17	14,04	73	18,03
0,9860	10,36	16	14,13	72	18,12
59	10,44	15	14,22	71	18,22
58	10,53	14	14,30		
57	10,61	13	14,39	0,9770	18,31
56	10,69	12	14,48	69	18,40
55	10,78	11	14,57	68	18,50
54	10,86			67	18,59
53	10,94	0,9810	14,66	66	18,69
52	11,03	09	14,75	65	18,78
51	11,11	08	14,84	64	18,88
		07	14,93	63	18,97
0,9850	11,19	06	15,02	62	19,07
49	11,28	05	15,11	61	19,16
48	11,36	04	15,20		
47	11,45	03	15,28	0,9760	19,26
46	11,53	02	15,37	59	19,35
45	11,61	01	15,46	58	19,45
44	11,70			57	19,54
43	11,78	0,9800	15,55	56	19,64
42	11,87	99	15,64	55	19,73
41	11,95	98	15,73	54	19,83
		97	15,82	53	19,92
0,9840	12,04	96	15,91	52	20,02
39	12,12	95	16,00	51	20,11
38	12,21	94	16,10		
37	12,29	93	16,19	0,9740	21,14
36	12,38	92	16,28	39	21,23
35	12,47	91	16,37	38	21,32
34	12,55			37	21,41
33	12,64	0,9790	16,46	36	21,50
32	12,73	89	16,55	35	21,60
31	12,81	88	16,64	34	21,69
		87	16,73	33	21,78
0,9830	12,90	86	16,83	32	21,87
29	12,99	85	16,92	31	21,96
28	13,07	84	17,01		
27	13,16	83	17,10	0,9730	22,05

Figure S1. Calculation table for the determination of ethanol content in fruit wines based on the density.

foods

MDPI

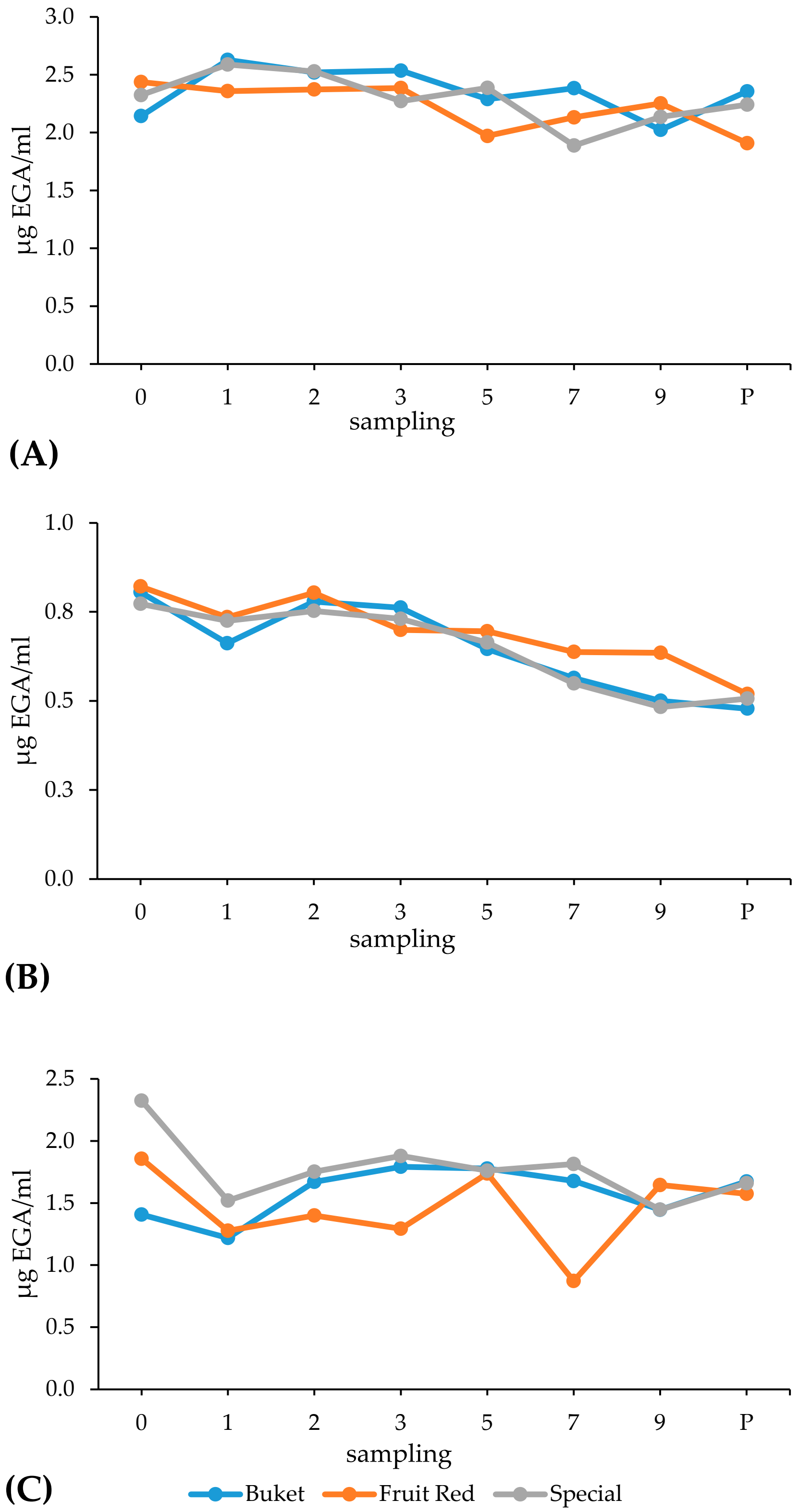

Figure S2. Total polyphenol content of hawthorn, apple and plum wines fermented by yeast strains Buket, Fruit Red and Special during fermentation and after pasteurization. A: hawthorn wine; B: apple wine; C: plum wine; EGA: equivalent of gallic acid; sampling: numbers represent days of sampling and letter „P“ means for sampling after pasteurization.

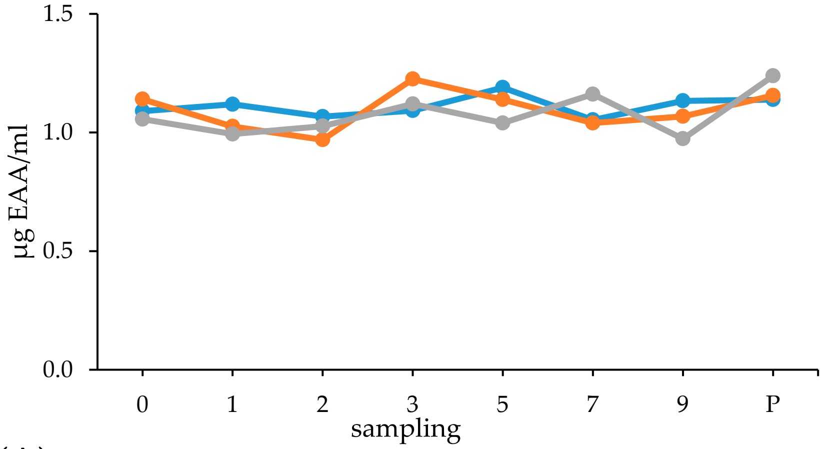

(A)

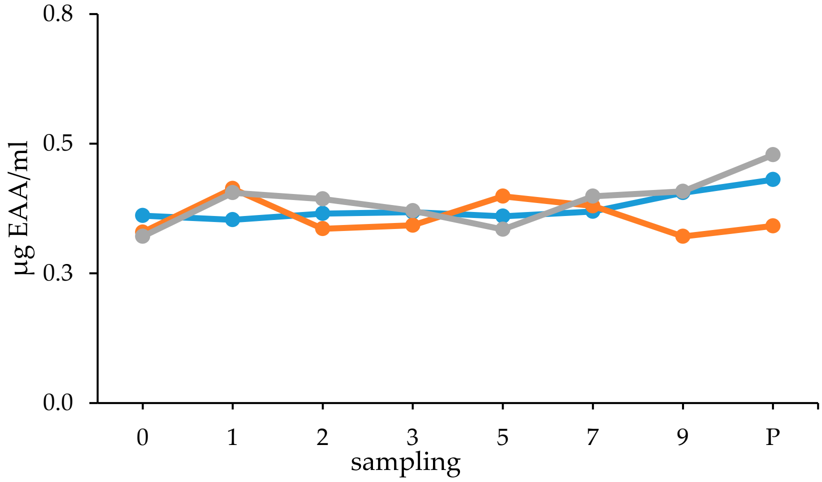

(B)

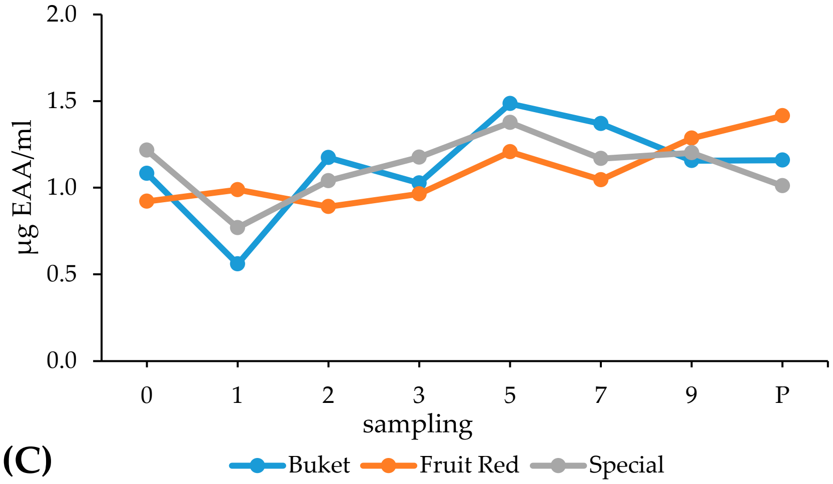

(C)

Figure S3. Development of scavenging activity of hawthorn, apple and plum wines fermented by yeast strains Buket, Fruit Red and Special during fermentation and after pasteurization against radical DPPH. A: hawthorn wine; B: apple wine; C: plum wine; EAA: equivalent of ascorbic acid; sampling: numbers represent days of sampling and letter „S“ means for sampling after pasteurization.

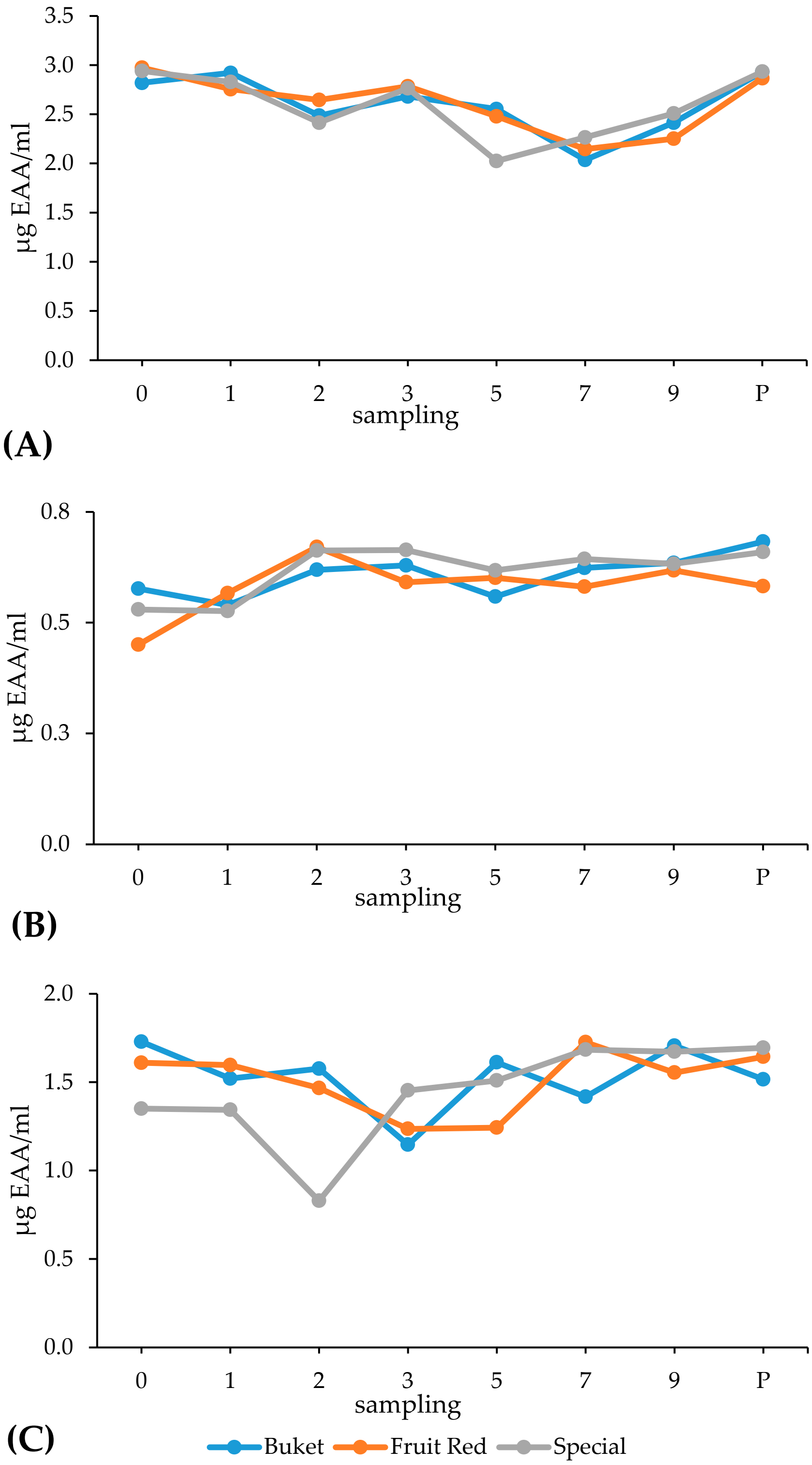

Figure S4. Development of scavenging activity of hawthorn, apple and plum wines fermented by yeast strains Buket, Fruit Red and Special during fermentation and after pasteurization against radical ABTS. A: hawthorn wine; B: apple wine; C: plum wine; EAA: equivalent of ascorbic acid; sampling: numbers represent days of sampling and letter „S“ means for sampling after pasteurization.

foods

MDPI

Table S1. Evaluation of color of hawthorn, apple and plum wines fermented by different yeast strains in the zero, ninth days and after pasteurization using CIELAB (L*a*b*) color space.

S	Y	L*			a*			b*		
		Hawthorn	Apple	Plum	Hawthorn	Apple	Plum	Hawthorn	Apple	Plum
	B	45.9±0.1^{Be}	57.4±0.04^{Ag}	41.2±0.21^{Cg}	37.7±0.12^{Ab}	8.6±0.01^{Ca}	32.2±0.27^{Bc}	29.2±0.17^{Ba}	45.3±0.06^{Aa}	27.0±0.35^{Ccd}
0	F	45.8±0.04^{Be}	58.0±0.19^{Af}	42.5±0.06^{Cf}	38.3±0.04^{Aa}	8.0±0.04^{Cb}	34.6±0.08^{Ba}	29.1±0.09^{Ba}	45.0±0.24^{Ab}	28.6±0.10^{Cb}
	S	45.5±0.12^{Bf}	57.8±0.06^{Af}	41.3±0.01^{Cg}	38.2±0.17^{Aa}	8.6±0.01^{Ca}	33.4±0.04^{Bb}	28.8±0.20^{Bb}	45.5±0.09^{A}a	26.9±0.02^{Cd}
	B	48.2±0.02^{Bb}	69.1±0.05^{Ab}	43.6±0.04^{Ce}	28.4±0.02^{Ag}	-0.9±0.01^{Ce}	28.2±0.02^{Bd}	20.7±0.00^{Cg}	25.7±0.09^{Be}	27.2±0.04^{Acd}
9	F	48.8±0.02^{Ba}	66.4±0.04^{Ae}	46.7±0.01^{Cb}	30.0±0.04^{Af}	0.9±0.00^{Cc}	26.5±0.01^{Bf}	23.9±0.04^{Cc}	31.6±0.04^{Ac}	30.5±0.00^{Ba}
	S	47.9±0.06^{Bc}	69.5±0.03^{Aa}	44.3±0.10^{Cd}	28.4±0.05^{Ag}	-1.2±0.01^{Bg}	28.2±0.12^{Ad}	20.5±0.04^{Cf}	24.0±0.02^{Bf}	26.9±0.15^{Ad}
	B	44.2±0.01^{Ch}	67.7±0.14^{Ad}	46.2±0.02^{Bc}	30.9±0.02^{Ac}	-0.5±0.00^{Cd}	27.3±0.03^{Be}	22.4±0.01^{Cd}	27.2±0.06^{Ad}	26.0±0.04^{Be}
P	F	47.4±0.02^{Cd}	68.4±0.10^{Ac}	47.9±0.15^{Ba}	30.3±0.02^{Ae}	-1.1±0.00^{Cf}	26.0±0.12^{Bg}	22.6±0.04^{Cd}	23.6±0.05^{Bg}	27.3±0.18^{Ac}
	S	44.8±0.01^{Cg}	68.4±0.02^{Ac}	46.1±0.02^{Bc}	30.7±0.01^{Ad}	-0.5±0.01^{Cd}	27.5±0.01^{Be}	21.9±0.01^{Ce}	27.1±0.02^{Ad}	26.3±0.01^{Be}

S: sampling day; Y: yeast, B: yeast „Buket“; F: yeast „Fruit Red“; S: yeast „Special“; results are presented as mean ± standard deviation (n = 3); $^{a–h}$ values with different superscripts (small letters) within a column differ significantly (p < 0.05) based on Tukey's HSD test. $^{A-C}$ values with different superscripts (big letters) within a row and single color component (L*, a* or b*) differ significantly (p < 0.05) based on Tukey's HSD test.

foods

MDPI

Table S2. Development of pH value of hawthorn, apple and plum wines fermented by different yeast strains during fermentation and after pasteurization.

S	Hawthorn wine			Apple wine			Plum wine		
	Buket	Fruit Red	Special	Buket	Fruit Red	Special	Buket	Fruit Red	Special
0	2.92±0,02^{Dbc}	2.90±0.03^{Dab}	2.90±0.01^{Dcde}	3.58±0.03^{Ac}	3.58±0.01^{Ade}	3.53±0.02^{Ad}	3.38±0.03^{Cc}	3.45±0.01^{Bb}	3.38±0.01^{Cc}
1	2.80±0,03^{Ce}	2.78±0.02^{Cc}	2.80±0.03^{Cf}	3.54±0.01^{Ac}	3.51±0.02^{Af}	3.54±0.01^{Ad}	3.31±0.01^{Bde}	3.32±0.01^{Bc}	3.29±0.04^{Bd}
2	2.82±0,03^{Cde}	2.79±0.04^{Cc}	2.83±0.03^{Cef}	3.56±0.02^{Ac}	3.54±0.00^{Aef}	3.56±0.00^{Ad}	3.28±0.00^{Be}	3.34±0.04^{Bc}	3.30±0.01^{Bd}
3	2.82±0,02^{Ede}	2.84±0.02^{Ebc}	2.85±0.02^{Edef}	3.67±0.02^{Ab}	3.61±0.02^{Bd}	3.61±0.02^{ABc}	3.34±0.02^{Dcd}	3.43±0.04^{Cb}	3.34±0.00^{Dcd}
5	2.88±0,01^{Dcd}	2.88±0.02^{Db}	2.91±0.03^{Dbcd}	3.69±0.01^{Ab}	3.70±0.01^{Ac}	3.66±0.01^{Ab}	3.51±0.03^{Cb}	3.60±0.02^{Ba}	3.51±0.02^{Cb}
7	2.94±0,04^{Dbc}	2.89±0.03^{Dab}	2.95±0.03^{Dbc}	3.77±0.03^{Aa}	3.73±0.01^{Abc}	3.78±0.01^{Aa}	3.54±0.02^{Cb}	3.62±0.02^{Ba}	3.53±0.03^{Cb}
9	2.97±0,02^{DEb}	2.91±0.04^{Eab}	2.98±0.02^{Dab}	3.77±0.01^{Aa}	3.76±0.01^{Ab}	3.77±0.02^{Aa}	3.55±0.02^{Cb}	3.64±0.03^{Ba}	3.55±0.03^{Cb}
P	3.04±0,02^{Ca}	2.96±0.02^{Da}	3.04±0.02^{Ca}	3.80±0.03^{Aa}	3.80±0.02^{Aa}	3.77±0.02^{Aa}	3.61±0.03^{Ba}	3.66±0.02^{Ba}	3.61±0.02^{Ba}

S: sampling day; numbers represent days of sampling and letter „P“ means for sampling after pasteurization; results are presented as mean ± standard deviation (n = 3); $^{a\text{-}f:}$ values with different superscripts (small letters) within a column and the same yeast differ significantly ($p < 0.05$) based on Tukey's HSD test; $^{A\text{-}E: :}$ values with different superscripts (big letters) within a row and the same sampling day differ significantly ($p < 0.05$) based on Tukey's HSD test.

foods

Table S3. Statistical analysis of significant differences between fruit wine types, yeasts, sampling and the combined effect of these factors within antioxidant potential parameters of pasteurized wines from hawthorn, apple and plum wines fermented by different yeast strains.

	TPC	DPPH	ABTS
Fruit	<0.001	<0.001	<0.001
Sampling	<0.001	<0.001	<0.001
Yeast	<0.001	0.257	<0.001
Fruit*Sampling	<0.001	<0.001	<0.001
Fruit*Yeast	<0.001	0.526	<0.001
Sampling*Yeast	<0.001	0.002	<0.001
Fruit*Sampling*Yeast	<0.001	<0.001	<0.001

Red numbers represent the statistically significant differences between samples ($p < 0.05$) based on multi-factor ANOVA.

foods

MDPI

Table S4. Statistical analysis of significant differences between fruit wine types, yeasts and the combined effect of fruit and yeast within sensory parameters of pasteurized wines from hawthorn, apple and plum wines fermented by different yeast strains.

	Color	Turbidity	Aroma	Overall taste	Sour taste intensity	Sweet taste intensity	Overall acceptability
Fruit	0.825	<0.001	0.204	0.031	0.016	0.001	0.024
Yeast	<0.001	0.002	0.661	<0.001	<0.001	<0.001	<0.001
Fruit*Yeast	0.063	0.147	0.156	0.006	0.008	<0.001	0.004

Red numbers represent the statistically significant differences between samples (p < 0.05) based on multi-factor ANOVA.

Table S5. Values of the sensory parameters (0 = dislike extremely, 10 = like extremely) of hawthorn, apple and plum wines fermented by different yeast strains after pasteurization.

Fruit	Yeast	Color	Turbidity	Aroma	Overall taste	Sour taste intensity	Sweet taste intensity	Overall acceptability
	B	6.7±2.5^{abc}	3.5±2.3^{abc}	6.0±2.2^{a}	4.3±2.1^{cde}	6.4±1.8^{a}	2.8±1.5^{bc}	3.9±1.9^{cde}
H	F	5.7±2.6^{bc}	5.1±3.1^{a}	5.0±2.1^{a}	7.3±2.1^{a}	3.2±1.5^{c}	6.8±1.6^{a}	7.1±1.9^{a}
	S	7.2±1.5^{ab}	3.9±2.5^{ab}	5.9±2.0^{a}	4.1±2.1^{cde}	6.7±1.9^{a}	2.9±1.8^{bc}	4.2±1.8^{cde}
	B	7.6±1.8^{a}	1.2±1.3^{d}	5.2±2.2^{a}	3.0±2.5^{de}	5.1±2.7^{ab}	1.8±1.6^{c}	3.1±2.3^{e}
A	F	5.0±2.4^{c}	3.6±2.5^{abc}	5.5±2.0^{a}	6.7±1.8^{ab}	3.6±2.0^{bc}	5.9±2.4^{a}	6.2±1.6^{ab}
	S	7.4±1.8^{ab}	2.0±1,9^{cd}	5.2±2.0^{a}	4.0±2.5^{de}	5.4±2.7^{ab}	1.7±1.3^{c}	3.6±2.1^{de}
	B	6.7±1.8^{abc}	3.0±2.0^{bcd}	5.2±2.9^{a}	5.4±2.8^{abcd}	5.9±2.2^{a}	3.2±1.7^{bc}	4.8±2.5^{bcde}
P	F	6.1±2.1^{abc}	3.2±1.6^{bc}	6.5±2.6^{a}	6.0±2.1^{abc}	5.2±1.9^{ab}	4.1±1.5^{b}	5.5±1.9^{abc}
	S	6.6±2.2^{abc}	2.9±1.5^{bcd}	6.2±2.6^{a}	5.2±2.4^{bcd}	5.9±2.4^{a}	3.9±2.2^{b}	5.1±2.5^{bcd}

H: hawthorn wine; A: apple wine; P: plum wine; B: yeast „Buket"; F: yeast „Fruit Red"; S: yeast „Special"; results are presented as mean ± standard deviation (n = 26); $^{a-e}$ values with different superscripts within a column and the same yeast differ significantly ($p < 0.05$) based on Tukey's HSD test.
